# Mitochondrial clearance by the STK38 kinase supports oncogenic Ras-induced cell transformation

**DOI:** 10.18632/oncotarget.9875

**Published:** 2016-06-07

**Authors:** Audrey Bettoun, Carine Joffre, Giulia Zago, Didier Surdez, David Vallerand, Ramazan Gundogdu, Ahmad A.D. Sharif, Marta Gomez, Ilaria Cascone, Brigitte Meunier, Michael A. White, Patrice Codogno, Maria Carla Parrini, Jacques H. Camonis, Alexander Hergovich

**Affiliations:** ^1^ Institut Curie, Inserm U830, Paris Sciences et Lettres University Paris, 75248, France; ^2^ University College London, Cancer Institute, London, WC1E 6BT, United Kingdom; ^3^ University of Texas Southwestern Medical Center, Dallas, Texas, 75390, USA; ^4^ Inserm U1151-CNRS UMR 8253, Institut Necker Enfants-Malades, Paris, 75993, France; ^5^ Present address: Cancer Research Center of Toulouse, UMR1037, Toulouse, 31100, France

**Keywords:** Ras GTPase, STK38, selective autophagy, mitophagy, cellular transformation

## Abstract

Oncogenic Ras signalling occurs frequently in many human cancers. However, no effective targeted therapies are currently available to treat patients suffering from Ras-driven tumours. Therefore, it is imperative to identify downstream effectors of Ras signalling that potentially represent promising new therapeutic options. Particularly, considering that autophagy inhibition can impair the survival of Ras-transformed cells in tissue culture and mouse models, an understanding of factors regulating the balance between autophagy and apoptosis in Ras-transformed human cells is needed. Here, we report critical roles of the STK38 protein kinase in oncogenic Ras transformation. STK38 knockdown impaired anoikis resistance, anchorage-independent soft agar growth, and *in vivo* xenograft growth of Ras-transformed human cells. Mechanistically, STK38 supports Ras-driven transformation through promoting detachment-induced autophagy. Even more importantly, upon cell detachment STK38 is required to sustain the removal of damaged mitochondria by mitophagy, a selective autophagic process, to prevent excessive mitochondrial reactive oxygen species production that can negatively affect cancer cell survival. Significantly, knockdown of PINK1 or Parkin, two positive regulators of mitophagy, also impaired anoikis resistance and anchorage-independent growth of Ras-transformed human cells, while knockdown of USP30, a negative regulator of PINK1/Parkin-mediated mitophagy, restored anchorage-independent growth of STK38-depleted Ras-transformed human cells. Therefore, our findings collectively reveal novel molecular players that determine whether Ras-transformed human cells die or survive upon cell detachment, which potentially could be exploited for the development of novel strategies to target Ras-transformed cells.

## INTRODUCTION

Cancer progression is subdivided into different major steps leading to malignant transformation and consequently the formation of aggressive tumours [1]. Two key steps and hallmarks of malignant cancer cells are resistance to anoikis (cell detachment-induced apoptosis [2]) and anchorage-independent growth [3]. Tumour cells use different strategies to circumvent anoikis [3]. For example, a compensatory strategy is the constitutive activation of pro-survival signals through pro-tumourigenic pathways such as Ras signalling, while an alternative strategy is the activation of cellular processes such as detachment-induced macroautophagy, which enables tumour cells to survive stress associated with extracellular matrix (ECM) detachment [4]. Whatever the mechanism to acquire anoikis resistance, the main consequence is the loss of anchorage-dependent growth, enabling detached tumour cells to proliferate, invade adjacent tissues, and hence potentially spread through the body [3].

Macroautophagy (henceforth termed autophagy) is a physiological mechanism for cell survival under stress conditions. During normal cell homeostasis, autophagy acts as a catabolic process to promote the degradation of potentially toxic protein aggregates and damaged organelles by encasing them in specialized intracellular autophagosomal vesicles for subsequent break down by lysosomal proteases for recycling [5]. Upon stress, the rate of autophagy increases to support survival, growth and metabolism [6]. Consequently, the modulation of autophagy is recognised as a hallmark of human cancer cells with context-dependent functions [7, 8]. Upon cancer initiation, autophagy may be tumour suppressive, while at advanced cancer stages autophagy tends to support malignant transformation by promoting survival of ECM-detached cancer cells [4, 9]. However, the signalling mechanisms controlling detachment-induced autophagy are not fully understood [4].

The small GTPase family of Ras proteins are frequently mutated proto-oncogenes in human cancers with a high prevalence in colorectal, pancreatic and lung cancers, where cancers carrying activating mutations in Ras are linked to particularly poor prognosis [10]. Thus, the selective targeting of downstream effectors of Ras signalling has seen substantial research investments. However, no effective targeted therapies currently exist for patients with tumours carrying mutant Ras. A recent example is MAPK pathway inhibition, where MEK inhibitor therapy is largely ineffective in individuals carrying oncogenic Ras mutations [11, 12]. Thus, there is a need to uncover molecular targets that potentially can be exploited to develop new therapeutic strategies for the targeted treatment of Ras-driven tumours. In this regard, mouse data indicate that tumours expressing oncogenic Ras can depend on autophagy for survival [13–18], suggesting that Ras-driven tumours can be “autophagy addicted” [13]. In support of this “autophagy addiction” model, it was reported that autophagy is required for robust anchorage-independent growth of Ras-transformed human cells [9, 13, 19, 20]. Thus, these preclinical studies suggest that autophagy is critical for malignant transformation through activated Ras, hence proposing that acute autophagy inhibition may be therapeutically beneficial in Ras-driven cancers. However, we currently do not fully understand whether the inhibition of a specific type of autophagy is sufficient to impair the survival of Ras-transformed cells. For example, it was reported that mitophagy, a selective autophagic processes targeting specifically mitochondria [21–23], can maintain the pool of functional mitochondria necessary to support growth of Ras-driven tumours [13, 24], while Ras-induced autophagy can mediate the functional loss of mitochondria during transformation [25]. Thus, the role of mitophagy in Ras-transformed human cells is incompletely understood.

Here, we report that the survival of Ras-transformed human cells is dependent on the STK38 (aka NDR1) serine/threonine protein kinase [26, 27]. More specifically, we define novel cancer cell biology roles of STK38 as a facilitator of pro-survival mechanisms in Ras-driven transformation. Considering that STK38 was recently linked as a pro-apoptotic kinase to possibly tumour suppressive activities in adherent cells [28–32], we had hypothesized that STK38 might play a role in opposing Ras-mediated malignant transformation by promoting apoptosis in stress conditions. However, to our surprise, we found that STK38 supports the survival of Ras-transformed human cells upon cell detachment. Given this observation and that our research recently revealed that STK38 positively promotes autophagy in adherent cells [29], we therefore postulated that STK38-mediated autophagy might support anoikis resistance and/or anchorage-independent growth in Ras-transformed cells. Indeed, STK38 knockdown resulted in diminished detachment-induced autophagy and anchorage-independent growth, paralleled by increased anoikis. We further found that STK38 potentially contributes to the survival of Ras-transformed human cells by supporting mitophagy. Knockdown of PINK1 or Parkin, the two best characterised regulators of mitophagy [22, 23], also impaired anoikis resistance and anchorage-independent growth of Ras-transformed human cells. Collectively, these findings uncover that regulators of mitophagy can determine whether Ras-transformed cells die or survive upon cell detachment, which could be exploited for the development of novel strategies to treat Ras-transformed cancer cells.

## RESULTS

### The STK38 kinase supports Ras-induced transformation of human cells

**Figure 1:**
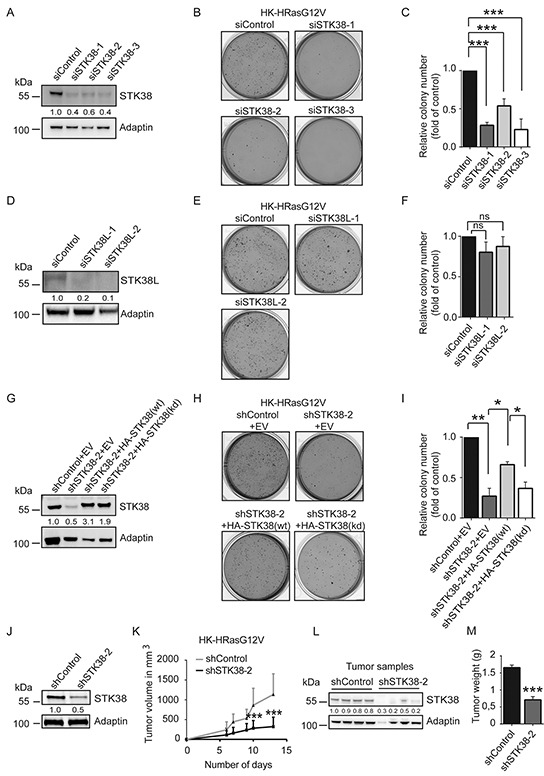
STK38 is required for anchorage independent growth and tumourigenicity of HRas-transformed human cells **A, D.** Immunoblotting with indicated antibodies of cell lysates derived from H-Ras^G12V^-transformed HK-HT cells (HK-HRasG12V) transiently transfected for 72 hrs with indicated siRNAs. Densitometry quantifications of immunoblots are indicated below the immunoblots. **B, C, E, F.** Depletion of STK38, but not STK38L, decreases anchorage-independent growth of HK-HRasG12V cells. Cells transiently transfected with indicated siRNAs were subjected to soft agar growth assays. Representative images of soft agar assays (B, E). Quantifications of colony formation in soft agar (C, F). The average of three experiments performed in duplicates is shown (n=3, ***p<0.001; ns, not significant). **G-I.** STK38 kinase activity is necessary for anchorage independent growth of HK-HRasG12V cells. (G) Western blot analysis of cells stably co-expressing indicated shRNAs with empty vector (EV), RNAi-resistant HA-tagged STK38 wild-type (wt) or kinase-dead (kd). Densitometry quantifications of immunoblots are indicated below the immunoblots (G). Cells were subjected to soft agar growth assays. Representative images (H). Quantification of colony formation in soft agar (I). The average of three experiments performed in duplicates is shown (n=3, *p<0.05; **p<0.01). **J-M.** STK38 is critical for the tumourigenicity of HK-HRasG12V cells. Immunoblotting of cells stably expressing indicated shRNAs (J). Densitometry quantifications of immunoblots are indicated below the immunoblots (J). Tumour formation of xenografts was monitored by volume changes (K). After animal euthanasia, STK38 depletion was analysed in tumour samples (L) and the tumour weight determined (M). Data represent the average of 12 mice per group (***p<0.001).

To understand whether STK38 plays a role in Ras-mediated transformation of human cells, we initially selected HK-HT cells, since they are a well-defined cell system for studying Ras-driven malignant transformation by soft agar colony formation and xenograft tumour growth [33–37]. As anticipated, HK-HT cells were transformed upon expression of oncogenic H-RasG12V as evidenced by growth in soft agar (Supplementary Figure S1). To test whether STK38 signalling plays a role in this setting, we assessed the consequences of STK38 depletion using three different siRNAs (Figure 1) and overexpression of hyperactive STK38-PIF (Supplementary Figure S1). This revealed that HK-HT cells expressing oncogenic HRasG12V (henceforth called HK-HRasG12V) with transient STK38 depletion displayed significantly decreased soft agar colony formation (Figure 1A, 1B, 1C), while expression of hyperactive STK38-PIF [38] was not sufficient to transform HK-HT cells (Supplementary Figure S1). Although human STK38L (aka NDR2) is similar to human STK38 [26, 27], efficient STK38L depletion in HK-HRasG12V had no effect on anchorage-independent growth (Figure 1D, 1E, 1F). To test whether STK38 kinase activity plays a role in supporting oncogenic Ras-mediated tumourigenic growth, we generated HK-HRasG12V cells stably expressing STK38-targeted shRNAs together with empty vector (EV), RNAi-resistant HA-STK38 wild-type (wt), or RNAi-resistant HA-STK38 kinase-dead (kd) (Figure 1G). As shown in Figure 1H and 1I, expression of HA-STK38(wt) substantially restored anchorage-independent growth, while cells expressing EV or HA-STK38(kd) did not compensate for the loss-of-function of STK38. These findings argue that STK38 kinase activity supports the anchorage-independent growth of Ras-transformed human cells. To validate whether STK38 also has a critical function in promoting Ras-induced tumourigenesis *in vivo*, we injected HK-HRasG12V cells stably expressing control or STK38-targeted shRNAs (Figure 1J) as xenografts into nude mice and monitored tumour formation (Figure 1K). This revealed that STK38-depleted tumours (Figure 1L) displayed markedly decreased growth as judged by volume (Figure 1K) and weight (Figure 1M). Taken together, our data presented in Figure 1 indicate that the STK38 kinase supports key oncogenic features of Ras-transformed HK-HT cells, namely colony formation in soft agar and *in vivo* xenograft tumour growth.

**Figure 2:**
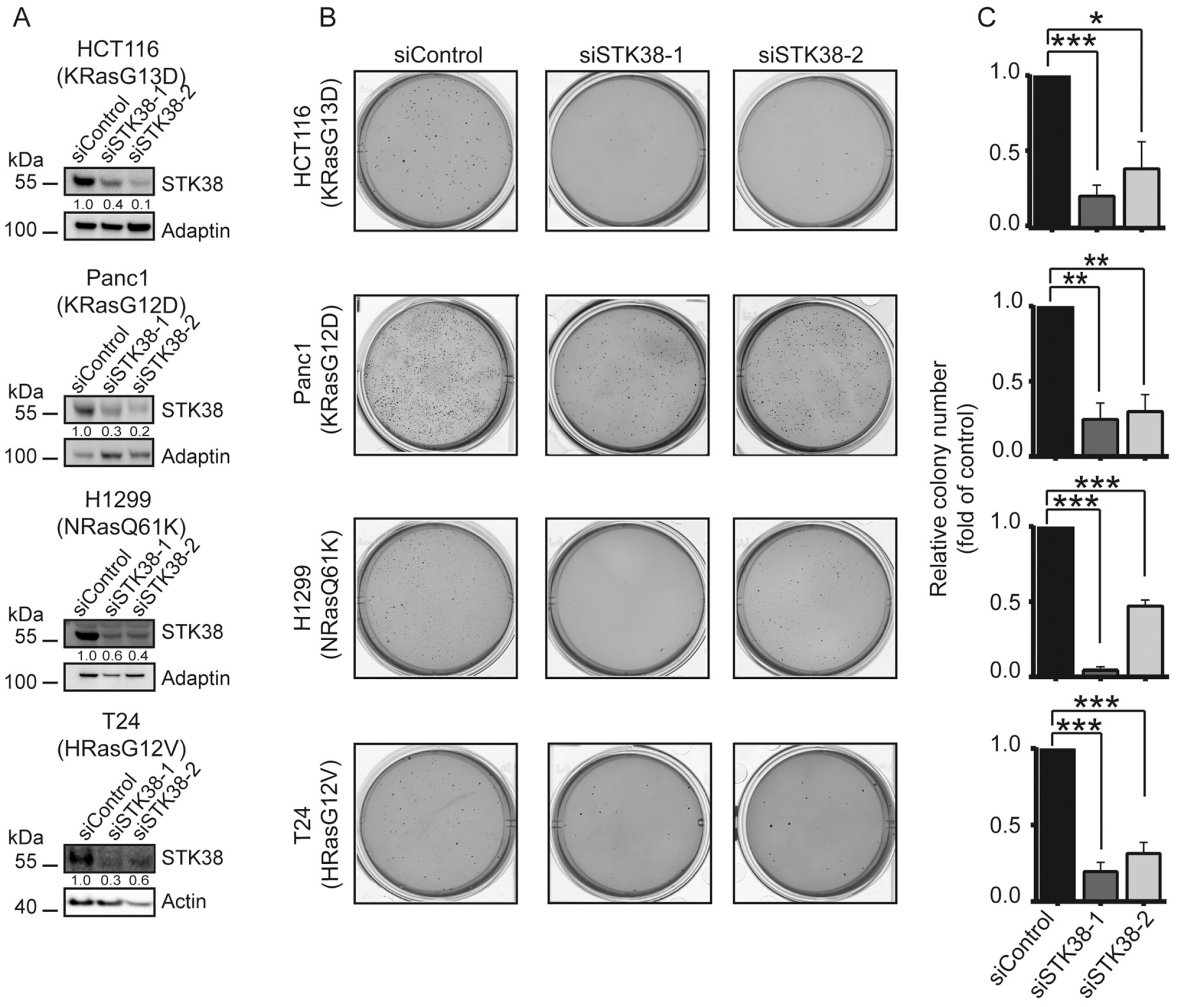
STK38 supports the anchorage independent growth of Ras-driven human cancer cell lines **A.** Western blotting with indicated antibodies of cell lysates transiently transfected for 72 hrs with indicated siRNAs. The mutation status of endogenous Ras isoforms is shown. Densitometry quantifications of immunoblots are displayed below the immunoblots. **B.** Cells transiently transfected with indicated siRNAs were subjected to soft agar growth assays. Representative images are displayed. **C.** Quantifications of colony formation in soft agar. The average of three experiments performed in duplicates is shown (n=3, *p<0.05; **p<0.01; ***p<0.001.

To investigate whether STK38 also promotes anchorage-independent growth of human cancer cells carrying endogenous activating mutations of Ras, we depleted STK38 in HCT116 (colorectal carcinoma cell line expressing KRas^G13D^), Panc1 (pancreatic adenocarcinoma cell line carrying KRas^G12D^), H1299 (lung carcinoma cell line expressing NRas^Q61K^), and T24 cells (urinary bladder carcinoma cell line carrying HRas^G12V^) (Figure 2). This revealed that STK38-depleted HCT116, Panc1, H1299 and T24 cells (Figure 2A) displayed significantly decreased anchorage-independent growth (Figure 2B, 2C), suggesting that STK38 plays a critical part in anchorage-independent growth of Ras-transformed human cells of diverse origins expressing different oncogenic Ras versions.

### p21/Cip1 upregulation in STK38-depleted Ras-transformed cells does not suppress anchorage-independent growth

**Figure 3:**
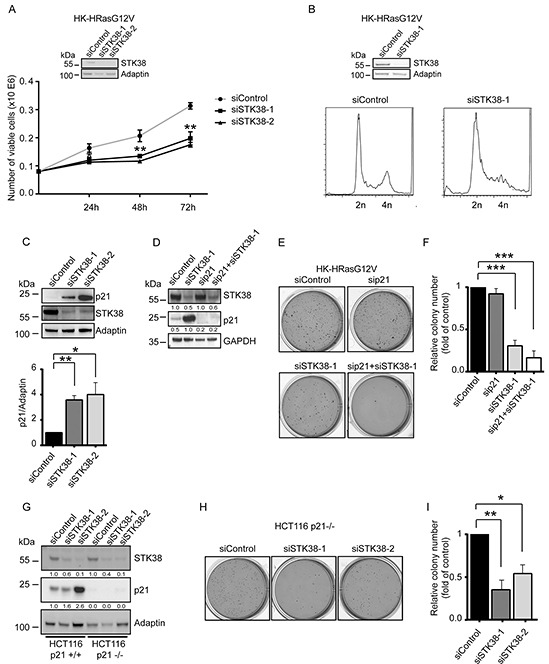
p21 upregulation in STK38-depleted Ras-transformed cells does not suppress anchorage-independent growth **A.** Proliferation rates of detached HK-HRasG12V cells transiently transfected with indicated siRNAs. The average of three experiments performed in duplicates is shown (n=3, **p<0.01). Validation of STK38 knockdown is shown as insert. **B.** Cell cycle analyses of detached HK-HRasG12V cells transiently transfected with indicated siRNAs. After 16 hrs in suspension, cells were processed for flow cytometry. One of three experiments is shown. Validation of STK38 depletion is shown as insert. **C.** Immunoblotting of HK-HRasG12V lysates transiently transfected with indicated siRNAs. Cells grew for 16 hrs in suspension before lysis. One of three experiments is shown (top panel). Densitometry quantification of immunoblots (bottom panel, n=3, *p<0.05; **p<0.01). **D-F.** Western blots of cells transiently transfected with indicated siRNAs (D). Densitometry quantifications of immunoblots are shown below the immunoblots (D). Cells were analysed by soft agar growth assays. Representative images (E). Quantification of colony formation in soft agar (F). The average of three experiments performed in duplicates is shown (n=3, ***p<0.001. **G-I.** Immunoblotting of cell lysates derived from parental and p21-null (p21−/−) HCT116 cells transiently transfected with the indicated siRNAs (G). Densitometry quantifications of immunoblots are specified below the immunoblots (G). Cells were subjected to soft agar growth assays. Representative images (H). Quantification of colony formation in soft agar (I). The average of three experiments performed in duplicates is shown (n=3, *p<0.05; **p<0.01).

In addition to soft agar growth (Supplementary Figure S1), HK-HRasG12V displayed anoikis resistance as judged by proliferation in suspension associated with reduced apoptosis upon cell detachment as assessed by PARP cleavage (Supplementary Figure S2A, S2B, S2C). STK38 depletion in HK-HRasG12V impaired proliferation in suspension (Figure 3A), accompanied by changes in the sub-G1, G1/S and G2/M cell cycle phases (Figure 3B). Considering that STK38 can regulate the G1/S transition through controlling p21^CDKN1A^ protein levels [39], we hypothesized that STK38 might support pro-survival oncogenic Ras signalling by suppressing p21 stabilisation. As anticipated [39], STK38 silencing in detached HK-HRasG12V resulted in significantly increased p21 protein levels (Figure 3C). However, upon knockdown of p21 together with STK38 in HK-HRasG12V (Figure 3D), soft agar growth was not increased when compared to cells with single knockdown of STK38 (Figure 3E and 3F). To consolidate this finding, KRasG13D-driven HCT116 cells with homozygous deletion of p21 [40] were analysed, revealing that STK38 depletion in p21-null HCT116 cells resulted in significantly reduced anchorage-independent growth (Figure 3G, 3H, 3I). Taken together, these observations suggest that STK38 can promote anchorage-independent growth of Ras-transformed cells independently of p21.

### STK38 supports detachment-induced autophagy and anoikis resistance in Ras-transformed human cells

**Figure 4:**
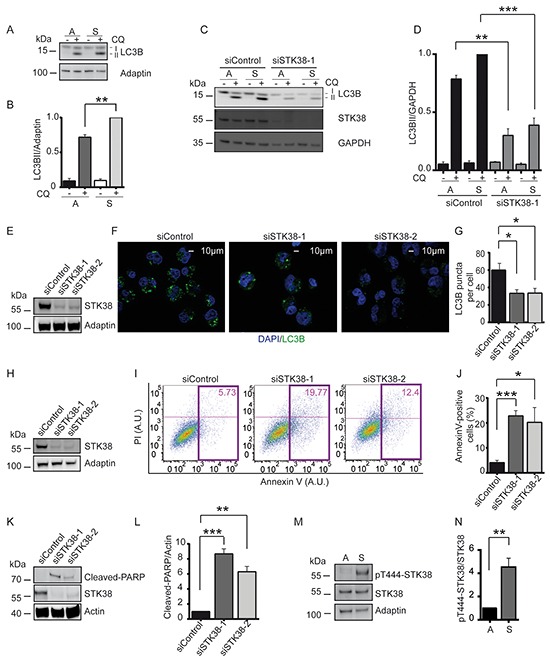
STK38 supports detachment-induced autophagy and anoikis resistance **A, B.** Immunoblot analysis of HK-HRasG12V cells grown in attached (A) or suspended (S) conditions with (+) or without (−) chloroquine (CQ). Lipidated (LC3B-II) and non-lipidated (LC3B-I) LC3B is indicated. One of three experiments is shown (A). Densitometry quantification of immunoblots (B, n=3, **p<0.01). **C, D.** Western blot analysis of cells transiently transfected with indicated siRNAs and grown in attached (A) or suspended (S) conditions with or without CQ. One of three experiments is shown (C). Densitometry quantification of immunoblots (D, n=3, **p<0.01; ***p<0.001). **E-G.** Immunoblots of cells transiently transfected with indicated siRNAs (E). Cells grown in suspension for 16 hrs in the presence of CQ were subjected to immunofluorescence analysis (F). LC3B is green. DNA is blue. Scale bar: 10 μm. Quantification of LC3 puncta per cell (G, at least 60 cells were analysed per condition, n=3, *p<0.05). **H-J.** Western blot analysis of cells transiently transfected with indicated siRNAs (H). Detached cells were processed for flow cytometry using AnnexinV-FITC and propidium iodide (PI) co-staining. One representative result is shown (I). Coloured rectangles and numbers indicate the number of apoptotic cells. Quantifications of apoptotic cells (J, n=3, *p<0.05; ***p<0.001). **K, L.** Immunoblot of detached cells transiently transfected with indicated siRNAs (K). One of three experiments is shown. Densitometry quantification of immunoblots (L, n=3, **p<0.01; ***p<0.001). **M, N.** Western blots of lysates derived from cells grown in attached (A) or suspended (S) conditions (M). Densitometry quantification of immunoblots (N, n=3, **p<0.01).

Considering that detachment-induced autophagy can promote anchorage-independent growth of Ras-transformed cells [4], we explored whether the role of STK38 as autophagy regulator [29] might contribute to anchorage-independent growth of Ras-transformed human cells. Similar as reported for HCT116, Panc1 and H1299 [9, 13, 20], we observed that HK-HRasG12V displayed elevated detachment-induced autophagy (Figure 4A, 4B, Supplementary Figure S2D, S2E) as assessed by conversion of LC3B-I to LC3B-II [41]. To visualise autophagic flux, LC3B was monitored in the presence of chloroquine (CQ), blocking lysosomal degradation of autophagosome contents. Detachment-induced changes of LC3B-II levels in HK-HRasG12V were at least in part Beclin1 dependent (Supplementary Figure S3). Moreover, in agreement with experiments studying other Ras-driven cancer cells [9, 13, 19, 20], genetic and pharmacological inhibition of autophagy in HK-HRasG12V resulted in decreased anchorage-independent growth (Supplementary Figure S4). Collectively, these findings demonstrate that HK-HRasG12V rely on autophagy for growth and survival in anchorage-independent conditions, hence establishing HK-HRasG12V as an excellent model system to explore the possible involvement of STK38-mediated autophagy in the survival and growth of detached Ras-transformed human cells.

As expected, STK38 depletion in adherent HK-HRasG12V cells resulted in reduced autophagy activity (Figure 4C, 4D) as evidenced by the LC3B-II conversion assay [41]. Significantly, detachment-induced autophagy was also diminished upon STK38 depletion in HK-HRasG12V (Figure 4C, 4D, Supplementary Figure S5). STK38 knockdown also reduced detachment-induced autophagy in HCT116 cells (Supplementary Figure S6A, S6B). As an alternative approach, LC3B puncta were analysed by immunofluorescence, an established readout for autophagosome formation [41], revealing that the number of autophagosomes was notably decreased upon STK38 knockdown (Figure 4E, 4F, 4G). Cumulatively, these data indicate that detachment-induced autophagy is mediated at least partly by STK38, and that this activity may protect Ras-transformed human cells from anoikis.

While HK-HT deprived of proper ECM contact underwent anoikis, HK-HRasG12V displayed anoikis resistance (Supplementary Figure S2A, S2B, S2C). Thus, we next tested whether STK38 promotes anoikis resistance in Ras-transformed cells. Intriguingly, detached STK38-depleted HK-HRasG12V displayed an increase in the sub-G1 cell cycle phase (Figure 3B), suggesting that STK38 depletion caused increased cell death. To examine whether this cell death was at least partly due to anoikis, we measured two independent markers of apoptosis, namely Annexin V staining by flow cytometry (Figure 4H, 4I, 4J) and PARP cleavage by immunoblotting (Figure 4K, 4L). Significantly, these analyses revealed that STK38-depleted HK-HRasG12V displayed dramatically elevated levels of anoikis when grown in suspension. Depletion of Beclin1, a key regulator of autophagy [42], also caused increased anoikis (Supplementary Figure S7) and interfered with anchorage-independent growth of HK-HRasG12V (Supplementary Figure S4). Considering that Beclin1 can function upstream of STK38 upon autophagy induction [29], these observations further strengthen the notion that STK38-mediated detachment-induced autophagy is critical to support oncogenic Ras signalling in order to antagonise anoikis and subsequently to promote anchorage-independent growth. In full agreement with this concept, we also detected considerably elevated STK38 kinase activity upon detachment of HK-HRasG12V cells (Figure 4M, 4N), as assessed by Thr444 phosphorylation of STK38, an established readout for STK38 kinase activity [27].

### Ral signalling supports detachment-induced autophagy and anoikis resistance in Ras-transformed human cells

**Figure 5:**
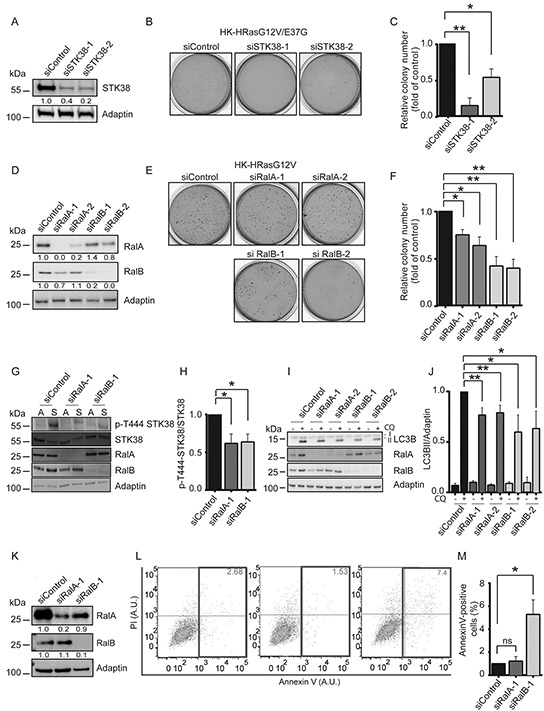
Ral GTPases support anchorage-independent growth and detachment-induced autophagy at least partly through STK38 as effector **A-C.** Immunoblotting of HK-HRasG12V/E37G lysates transiently transfected with indicated siRNAs (A). Densitometry quantifications of immunoblots are indicated below the immunoblots (A). Cells were tested by soft agar growth assays. Representative images (B). Quantification of colony formation in soft agar (C). The average of three experiments performed in duplicates is shown (C, n=3, *p<0.05; **p<0.01). **D-F.** Immunoblotting of HK-HRasG12V lysates transiently transfected with indicated siRNAs (D). Densitometry quantifications of immunoblots are shown below the blots (D). Cells were analysed by soft agar growth assays. Representative images (E). Quantification of colony formation in soft agar (F). The average of three experiments performed in duplicates is shown (F, n=3, *p<0.05; **p<0.01). **G, H.** Immunoblotting of lysates transiently transfected with indicated siRNAs and grown in attached (A) or suspended (S) conditions (G). Densitometry quantification of immunoblots (H, n=3, *p<0.05). **I, J.** Western blots of lysates of HK-HRasG12V cells transiently transfected with indicated siRNAs and grown in suspension with or without CQ (I). Densitometry quantification of immunoblots (J, n=3, *p<0.05; **p<0.01). **K-M.** Immunoblotting of cells transiently transfected with indicated siRNAs (K). Densitometry quantifications of immunoblots are indicated below the blots (K). Cells were grown in suspension for 16 hrs before processing for flow cytometry using AnnexinV-FITC and propidium iodide (PI) co-staining. One representative result is shown (L). Rectangles and numbers indicate the number of apoptotic cells. Quantifications of the percentage of apoptotic cells (M, n=3, *p<0.05; ns, not significant).

Ral-Exocyst signalling promotes anchorage-independent growth in HK-HRasG12V cells [34–37] and supports autophagy in adherent cells [43]. Therefore, we postulated that STK38 might act in Ras-mediated transformation by functioning downstream of Ras-Ral-Exocyst signalling. To test this idea, we pursued different experimental avenues. First, we tested STK38 depletion in HK-HT expressing a HRasG12V/E37G mutant [44], which mainly activates RalGEFs, while only weakly stimulating Raf or PI3-kinase signalling [34] (Supplementary Figure S8A, S8B, S8C). As anticipated [34], the HRasG12V/E37G mutant transformed HK-HT (Supplementary Figure S8D, S8E). Noteworthy, STK38 depletion in HK-HRasG12V/E37G (Figure 5A) reduced growth of colonies in soft agar (Figure 5B, 5C), suggesting that STK38 can function downstream of Ral signalling. Secondly, depletion of RalA or RalB in HK-HRasG12V, which appears to affect both Ral proteins to some degree (Figure 5D), confirmed their involvement in anchorage-independent growth (Figure 5E, 5F). Thirdly, knockdown of the Exocyst components Sec5 or Exo84 in HK-HRasG12V also indicated that the Exocyst complex is critical to sustain anchorage-independent growth (Supplementary Figure S9). Taken together, these data demonstrate that Ral-Exocyst signalling significantly contributes to Ras-mediated transformation in HK-HT cells.

Next, to further examine whether STK38 acts downstream of Ral signalling, we investigated the consequences of RalA or RalB depletion on Thr444 phosphorylation of STK38, detachment-induced autophagy, and anoikis resistance. RalA or RalB knockdown in HK-HRasG12V significantly decreased detachment-induced Thr444 phosphorylation of STK38 (Figure 5G, 5H). Similar to results obtained with STK38-depleted cells (Figure 4C, 4D, Supplementary Figure S5), LC3B-II conversion assays showed reduced detachment-induced autophagy in RalA- or RalB-depleted HK-HRasG12V (Figure 5I, 5J). RalB depletion seems to lead to increased anoikis (Figure 5K, 5L, 5M), although to a much lesser degree than observed upon STK38 knockdown (Figure 4H, 4I, 4J). In contrast, RalA depletion appears to have no detectable effect on anoikis resistance (Figure 5K, 5L, 5M), although these effects of single RalA or RalB depletions have to be interpreted with caution, since they may affect the levels of both Ral proteins in our settings (Figure 5K). In summary, these data suggest that Ral signalling at least partly supports Ras-driven transformation through the STK38 kinase as a downstream effector.

### STK38 is required for mitochondrial clearance in Ras-transformed human cells

**Figure 6:**
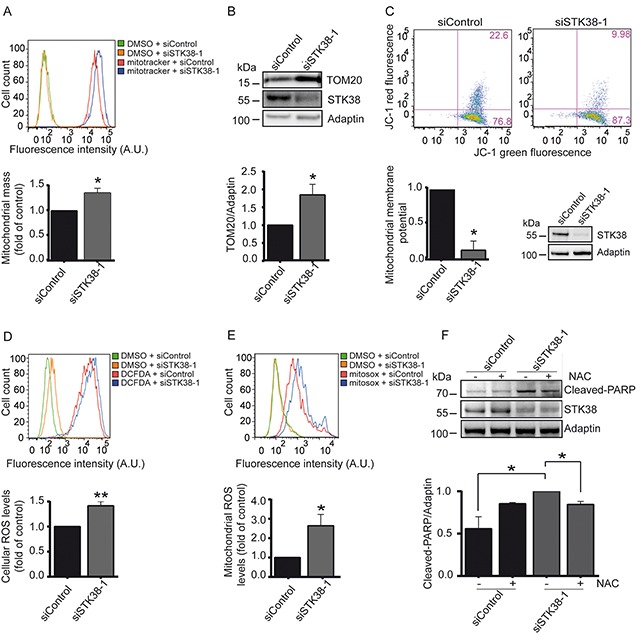
STK38 supports the clearance of damaged mitochondria to prevent ROS-mediated anoikis in detached Ras-transformed human cells **A-E.** Cells transiently transfected with indicated siRNAs and grown in suspension were subjected to flow cytometry (A, C, D, E) and immunoblotting (B, C) using indicated dyes and antibodies. One of three experiments is shown. Quantifications of total mitochondrial mass (A), densitometry of immunoblots (B), mitochondrial membrane potential (C), intracellular ROS levels (D) and mitochondrial ROS levels (E). (A-E, n=3, *p<0.05; **p<0.01). Validation of STK38 knockdown is shown as insert (C) or is presented in Supplementary Figures S10A and S10B. **F.** Cells were transiently transfected with indicated siRNAs and subsequently grown in suspension in the absence (−) or presence (+) of N-acetylcysteine (NAC), followed by processing for immunoblotting. One of three experiments is shown (top panel). Quantifications of densitometry of immunoblots (bottom panel, n=3, *p<0.05).

The main cancer-promoting roles of autophagy are the preservation of organelle function and the provision of energy substrates. Current evidence suggests that inhibition of mitophagy, which selectively targets damaged (depolarized) mitochondria for degradation by autophagy [23], can promote tumour progression and does not phenocopy inhibition of autophagy [22]. However, like autophagy, mitophagy may have context-dependent roles in cancer. For example, mitophagy maintains the pool of functional mitochondria necessary to support growth of Ras-driven tumours [13, 24], while Ras-induced autophagy can mediate the functional loss of mitochondria during transformation [25]. In this regard, it is intriguing that STK38 can act in mitochondrial quality control [45] in addition to its role in non-selective autophagy [29]. However, whether the STK38-mediated mitochondrial quality control pathway plays any role in cellular transformation is currently unknown. Thus, we tested whether STK38 can play a role in mitophagy in detached Ras-transformed human cells by studying different mitochondrial parameters (Figure 6A, 6B, 6C, Supplementary Figure S6, S10). First, we measured changes in mitochondrial mass using a membrane potential-independent MitoTracker (Figure 6A, Supplementary Figure S6C, S10A, S10C, S10D). Second, the levels of the non-respiratory outer mitochondrial membrane protein TOM20 and five inner mitochondrial membrane proteins were analysed (Figure 6B, Supplementary Figure S6D, S10E, S10F). Third, we used the JC-1 dye as an indicator of mitochondrial membrane potential (Figure 6C, Supplementary Figure S10G). These measurements collectively revealed that STK38-depleted Ras-transformed human cells displayed increased mitochondrial mass associated with decreased mitochondrial membrane potential, suggesting that in Ras-transformed cells STK38 is required to ensure the removal of damaged mitochondria. To confirm that the observed changes in mitochondrial mass are a consequence of decreased mitophagy, we pharmacologically and genetically inhibited autophagy. This revealed that autophagy inhibition by either CQ treatment or ATG5 knockdown resulted in significantly increased mitochondrial mass in detached HK-HRasG12V (Supplementary Figure S11A, S11B). Moreover, PINK1 depletion in detached HK-HRasG12V cells caused impaired mitophagy (Supplementary Figure S11C), while autophagy in general was not affected as judged by LC3B-II conversion assays (Supplementary Figure S12).

Noteworthy, upon cell detachment STK38-depleted HK-HRasG12V cells (Supplementary Figure S10B) displayed significantly elevated levels of reactive oxygen species (ROS) (Figure 6D), with mitochondrial ROS being considerably elevated (Figure 6E). Thus, we tested next whether elevated ROS production due to STK38 depletion caused increased cell death of Ras-transformed human cells. To do so, detached STK38-depleted HK-HRasG12V and HCT116 cells were treated with the ROS inhibitor N-acetyl-L-cysteine (NAC) and cell death was monitored by PARP cleavage (Figure 6F, Supplementary Figure S6E, S6F). Significantly, NAC treatment of STK38-depleted HK-HRasG12V and HCT116 cells at least in part decreased PARP cleavage in Ras-transformed human cells. Taken together, these findings suggest that STK38 supports the removal of damaged mitochondria by mitophagy to limit potentially toxic ROS production in detached Ras-transformed cells, which in turn can promote anoikis resistance and anchorage-independent growth of Ras-transformed cells.

Considering that our data (Figure 5, Supplementary Figure S8) indicate that the Ral pathway supports Ras-mediated transformation through STK38 as a downstream effector, we wondered next whether Ral signalling contributes to mitophagy in Ras-transformed cells. Thus, we monitored changes in mitochondrial mass upon knockdown of these signalling components in detached HK-HRasG12V cells (Supplementary Figure S13). This revealed that RalB-depleted cells displayed a significant increase in mitochondrial mass in contrast to control and RalA-depleted cells (Supplementary Figure S13). These findings suggest that RalB signalling contributes to mitophagy in Ras-transformed cells, while RalA signalling appears to be dispensable for mitophagy.

**Figure 7:**
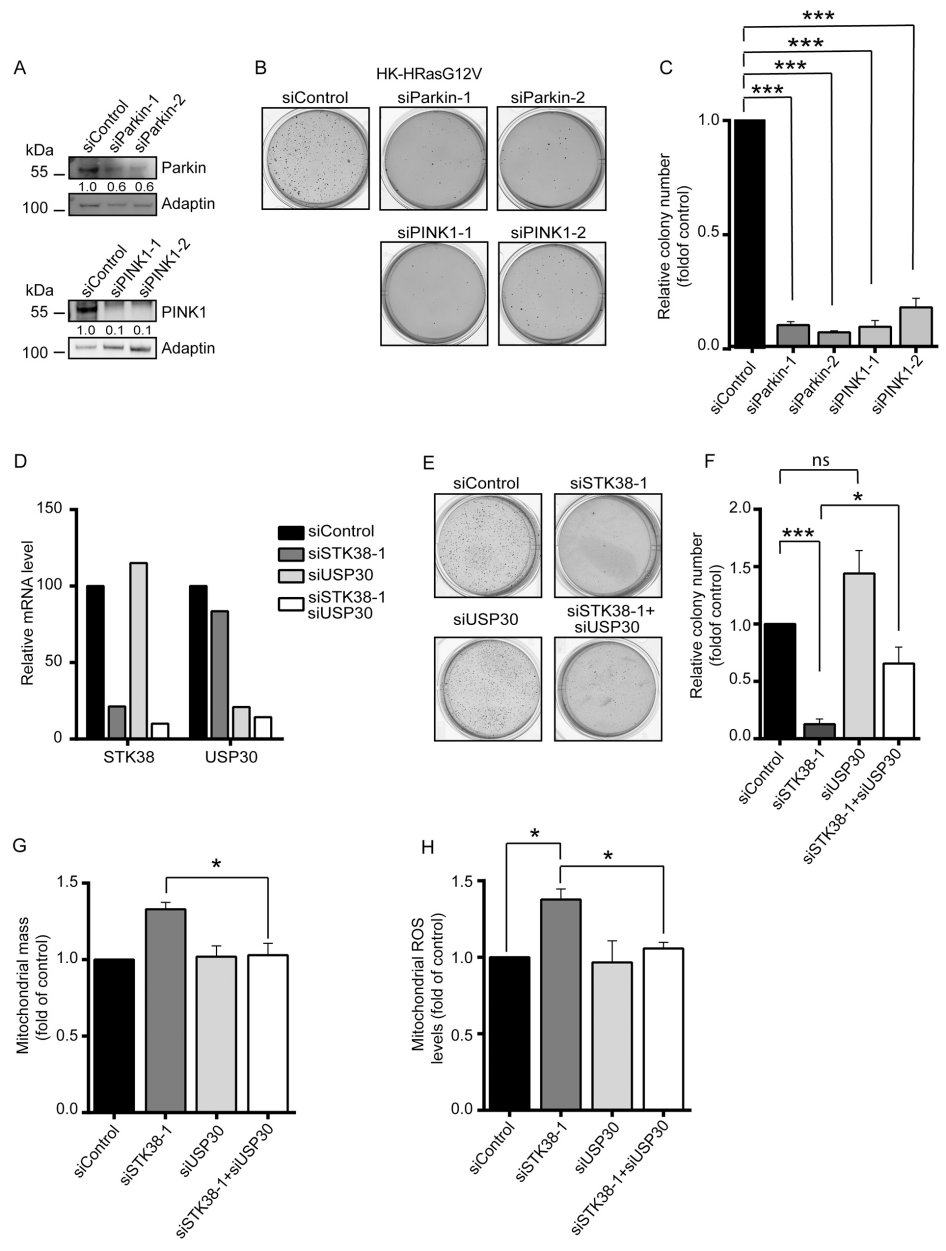
The mitophagy regulators Parkin, PINK1 and USP30 are linked to STK38 in supporting anchorage-independent growth of Ras-transformed human cells **A-C.** Western blot analysis of HK-RasG12V cells transiently transfected with indicated siRNAs (A). Densitometry quantifications of immunoblots are indicated below the immunoblots (A). Cells were subjected to soft agar growth assays. Representative images of soft agar assays are shown (B). Histograms represent the quantification of colony formation in soft agar (C). Average of three independent experiments performed in duplicates is shown (C, n=3, ***p<0.001). **D-F.** Real-time quantitative PCR analysis of STK38 and USP30 mRNA expression in HK-RasG12V cells transiently transfected with indicated siRNAs. The averaged results of triplicates are shown (D). Cells were subjected to soft agar growth assays. Representative images of soft agar assays are shown (E). Histograms represent the quantification of colony formation in soft agar (F). Average of three independent experiments performed in duplicates is shown (F, n=3, *p<0.05; ***p<0.001; ns, not significant). **G, H.** Cells transiently transfected with indicated siRNAs and grown in suspension were subjected to flow cytometry using a membrane potential-independent MitoTracker (G) or the MitoSOX Red dye (H). Histograms show the quantifications of total mitochondrial mass (G) and mitochondrial ROS levels (H) from three independent experiments (n=3, *p<0.05).

Finally, we wondered whether, similar to STK38, other regulators of mitophagy also support the survival of Ras-transformed human cells. Specifically, since mitophagy is regulated in many cell types by Parkin and PINK1 [22, 23] and loss of PINK1 can impair cancer-associated phenotypes including anchorage-independent growth of HeLa cells [46], we speculated that PINK1 and/or Parkin might play a role in promoting soft agar growth of Ras-transformed human cells. Indeed, PINK1 or Parkin depletion in HK-HRasG12V dramatically reduced soft agar growth, associated with increased anoikis as judged by elevated PARP cleavage (Figure 7A, 7B, 7C, Supplementary Figure S14A, S14B). Therefore, our data collectively suggest that the STK38 and PINK1/Parkin mitochondrial quality control pathways play an important role in Ras-induced malignant transformation.

To test whether the STK38 and PINK1/Parkin pathways are genetically linked in our settings, we studied the consequences of STK38 depletion combined with knockdown of USP30, a negative regulator of mitophagy that opposes PINK1/Parkin-mediated mitophagy [47, 48]. More precisely, the USP30 deubiquitinase can inhibit the mitophagic process by opposing the ubiquitination of mitochondrial proteins by the Parkin E3 ubiquitin ligase. In this regard, we speculated that USP30 depletion might compensate for STK38 deficiency, hence restoring anchorage-independent growth of STK38-depleted Ras-transformed cells. This final set of experiments showed that the knockdown of USP30 alone (Figure 7D) had no significant effect on the anchorage-independent growth of HK-HRasG12V cells (Figure 7E, 7F). Importantly, co-depletion of STK38 and USP30 (Figure 7D) resulted in a partial restoration of anchorage-independent growth of Ras-transformed human cells when compared to single STK38 knockdown (Figure 7E, 7F), suggesting that the STK38 and PINK1/Parkin/USP30 pathways can at least in part be interlinked in the context of the survival of Ras-transformed human cells. In this regard, we further observed that co-depletion of STK38 and USP30 was sufficient to fully restore total mitochondrial mass (Figure 7G) and mitochondrial ROS levels (Figure 7H) to normal values as observed in control cells. Taken together, these data indicate that mitophagy in general appears to be important for the survival of detached Ras-transformed human cells, with STK38 and established regulators of mitophagy supporting the anchorage-independent growth of Ras-transformed cells.

## DISCUSSION

Here, we report STK38 as a key mediator of Ras-driven transformation of human cells. STK38 knockdown causes impaired anoikis resistance, anchorage-independent soft agar growth, and xenograft *in vivo* growth of Ras-transformed cells (Figure 1, 2, 3). STK38 supports autophagy and mitophagy in detached Ras-transformed cells (Figure 4, 6), thereby promoting cancer cell survival by facilitating anoikis resistance. The Ral-Exocyst-STK38 pathway promotes anchorage-independent growth downstream of oncogenic Ras (Figure 1, 5, Supplementary Figure S8, S9). Specifically, RalA and RalB are at least in part critical for detachment-induced STK38 activation, detachment-induced autophagy, and anchorage-independent growth of Ras-transformed cells (Figure 5). However, only RalB appears to be important for anoikis resistance and efficient clearance of mitochondria in detached cells (Figure 5, Supplementary Figure S13). Thus, RalA and RalB seem to have distinct roles in Ras-driven transformation using STK38 as a downstream effector. Therefore, how the RalB-STK38 axis is directly regulated by upstream factors, and how STK38 connects Ras signalling to downstream effectors deserves future investigation, in order to further expand our understanding of the autophagic function(s) of STK38 in Ras-transformed cells.

Little is known about the complex crosstalk between anoikis and detachment-induced autophagy [3, 4]. Typically, autophagy antagonises apoptosis, and apoptosis induction reduces autophagy. In line with this general model, our data indicate that upon loss of ECM-cell contact STK38 seems to function as a suppressor of anoikis while promoting detachment-induced autophagy (Figure 4). Considering that in adherent human cells STK38 can act as a pro-apoptotic kinase [28–32], this cytoprotective role of STK38 was unanticipated. Instead of defining, as initially anticipated, to which degree STK38 as a pro-apoptotic kinase opposes Ras-driven transformation, our study uncovered an unexpected pro-survival role of STK38 as a promoter of mitophagy. Thus, future studies of how STK38 is regulated in this context will help to understand how STK38 can switch from a pro-apoptotic role to a pro-survival function. In this regard, changes in the subcellular localisation patterns and/or regulatory binding partners of STK38 may play a role [27].

In further support of a role for STK38 as a pro-survival factor following ECM detachment, detached STK38-depleted Ras-transformed cells displayed loss of mitochondrial membrane potential (Figure 6, Supplementary Figure S10), suggesting that STK38 can prevent the accumulation of depolarised mitochondria. Therefore, we studied different mitochondrial parameters (Figure 6, Supplementary Figure S6, S10, S11), revealing that STK38 appears to be important for the removal of damaged mitochondria by mitophagy in Ras-transformed cells. In this regard, PINK1- or Parkin-depleted Ras-transformed cells also displayed decreased anoikis resistance and anchorage-independent growth (Figure 7, Supplementary Figure S14), while detachment-induced autophagy in general appeared to be unaffected by PINK1 knockdown (Supplementary Figure S12), which suggests that mitophagy is critical for anoikis resistance and potentially tumour formation. Even more importantly, depletion of USP30, a major opponent of PINK1/Parkin-mediated mitophagy [47, 48], partially restored soft agar growth, and fully restored total mitochondrial mass and ROS levels of STK38-depleted Ras-transformed cells (Figure 7). These findings indicate that the STK38 and PINK1/Parkin/USP30 pathways are potentially connected. Based on our findings it is likely that these pathways support the survival of Ras-transformed human cells by ensuring the removal of damaged mitochondria, which are prone to produce potentially toxic mitochondrial ROS [49, 50]. In this regard, our data (Figure 6, Supplementary Figure S6) further propose that STK38-mediated removal of damaged mitochondria can play a role in preventing the increased production of ROS, which has the potential to significantly decrease cancer cell survival [22]. Moreover, by defining STK38 as a potential regulator of mitophagy, our study lays a foundation for follow up studies to increase our understanding of how mitophagy is initiated and executed as well as how mitophagy can play a role in Ras-driven tumours. Thus, future research into these different aspects is warranted.

Cancer cell metabolism generally prefers aerobic glycolysis over mitochondrial oxidation, a phenomenon known as the Warburg effect [51]. In particular, cells in suspension increase glycolysis over glucose oxidation to meet their energy needs, and thereby contribute to anoikis resistance and the metastatic potential of cancer cells [52]. As a result, it is possible that STK38-mediated autophagy might help to meet the metabolic demands of Ras-transformed cells to facilitate the survival of tumour cells. Several observations would be supportive of this hypothesis: first, detachment-induced autophagy is critical for the survival in suspension [3, 9], second, inhibition of autophagy in Ras-driven cancer models can cause defective mitochondrial metabolism [4], and third, Ras signalling can re-programme cellular metabolism [53]. Consequently, much remains to be learned concerning the precise mechanisms through which autophagy controls metabolism [4]. In this context, future research may reveal that STK38-mediated autophagy can play a critical role in the enhanced glycolytic capacity of Ras-transformed cells, which would help to understand how autophagy and glycolysis are linked. Since autophagy is upregulated during metastasis [4, 54], our findings further propose that STK38-mediated detachment-induced autophagy might contribute to metastasis by promoting anoikis resistance, which can facilitate tumour cell dissemination and dormancy [55]. Considering further that apoptotic resistance is indispensable for all steps of metastatic progression with anoikis resistance being the most critical step [55], future research in suitable animal models is warranted to examine whether and how STK38-mediated autophagy can support metastasis. Such studies may help to develop a clear rationale for if (and when) pharmacological STK38 inhibition can interfere with metastasis.

In general, autophagy studies represent a research area with increasing interest regarding oncogenesis and cancer therapy resistance [4, 7, 8]. Thus, the definition of the context specific roles for autophagy in cancer and the regulatory signalling mechanisms is important to guide autophagy-based therapeutic intervention [8]. Particularly, the “autophagy addiction” of Ras-driven cancers might be exploitable by targeting autophagy. Thus, our discovery of STK38 as autophagy regulator in Ras-transformed cells may help to develop an intervention opportunity for Ras-driven cancers. As a protein kinase [26, 27], STK38 represents a potentially interesting drug target to inhibit autophagy activity in Ras-driven cancers. Considering further that STK38 knockout mice are viable and fertile with normal life spans [28, 56] and STK38 displays unique features in its regulatory and catalytic domains [26, 27], we propose that the development of pharmacological compounds that selectively target STK38 should be a feasible option for clinical approaches aiming to target “autophagy addicted” cancers. However, STK38 inhibitors will require stringent evaluation, since STK38 has other cellular functions [27] besides the roles described here, and STK38-deficiency can impair the immune system of mice [28, 57, 58]. Thus, it will be interesting to ascertain in appropriate preclinical cancer models whether selective and acute STK38 inhibition can be of therapeutic benefit to target Ras-driven cancer cells without harming healthy tissues.

Taken together, we define here STK38 as a facilitator of oncogenic Ras-driven transformation. STK38-mediated autophagy supports “autophagy addicted” Ras-transformed cells by sustaining anoikis resistance and anchorage-independent growth through functioning as effector of Ras-Ral signalling. Therefore, we provide here insights into a new signalling mechanism that could possibly be exploited for the development of novel clinical compounds allowing the targeted inhibition of autophagy in Ras-driven cancers. In this framework, our findings now draw attention to the importance of further understanding the selective vs. non-selective autophagic function(s) of STK38 and their upstream regulators and downstream effectors in a context-dependent manner.

## MATERIALS AND METHODS

### Tissue cell culture and drug treatments

Cells were cultured at 37°C and 5% CO2 in humidified chambers. HK-HT cells were grown in DMEM (Gibco) with 10% FBS (Gibco), 1% penicillin/streptomycin (Invitrogen),100 μg/ml hygromycin and 400 μg/ml neomycin [33]. Media for HK-HRasG12V and HK-HRasG12V/E37G cell pools were added 300 μg/ml zeocin. HK-HA-STK38-PIF, HK-HA-STK38-PIF/kd cell pools were added 1 μg/ml puromycin, HK-HRasG12V cell pools stably expressing shRNAs were added zeocin and puromycin, and HK-HRasG12V cell pools stably expressing shRNAs and RNAi-resistant HA-STK38 were added zeocin, puromycin, and 5 g/ml blasticidin. HCT116, Panc1, H1299, and T24 cells were from ATCC and cultured in DMEM with 10% FBS and 1% penicillin/streptomycin, except H1299 were grown in RPMI 1640 GlutaMAX (Invitrogen) instead of DMEM. Antibiotics were from Invivogen unless otherwise stated. For suspension culture, cells were grown attached to sub-confluence, before culturing in complete medium at 3 × 10 E5 cells per well on 0.02% pluronic acid-coated Ultra Low Attachment Surface 6-well plates (Corning). Chloroquine (CQ), 3-methyladenine (3-MA) and N-acetylcysteine (NAC) were from Sigma.

### Generation of stable cell lines

For retrovirus production HEK293Tv packaging cells were transfected with retroviral vectors and the packaging plasmid pcl10A1 (NBP2-29542, Novus) using Fugene 6 (Promega) according to manufacturer's instructions. 48 hrs post-transfection viral supernatants were harvested, filtered, and added to the recipient cell lines in the presence of 4 microgram per ml were used, so please put the “micro” symbol back. polybrene (Sigma-Aldrich). 24 hrs post-infection, recipient cells were selected with appropriate antibiotics.

### siRNA transfections

Cells were reverse transfected with siRNAs using Lipofectamine RNAiMax (Invitrogen) according to manufacturer's instructions. 72 hrs post-transfection, cells were harvested for immunoblotting or treated as defined in the specific sections.

### Source and sequences of siRNAs

siControl non-targeting siRNA (D-001810-01-50), siATG5-1 ON-TARGET and siATG5 smart pool (L-004374-00-0005) were purchased from Dharmacon. The remaining siRNAs were purchased from Eurogentec:

STK38-1: 5′- CCTTATCGCTCAACATGAAdTT-3′
STK38-2: 5′-CGTCGGCCATAAACAGCTAdTT-3′
STK38-3: 5′ GAGCAGGTTGGCCACATTCdTT-3′
STK38L-1: 5′-GTTACGTCGATCACAACACdTT-3′
STK38L-2: 5′-GACACCTTGACAGAAGAGGdTT-3′
RalA-1: 5′-GAGACAACTACTTCCGAAGdTT-3′
RalA-2: 5′-GACAGGTTTCTGTAGAAGAdTT-3′
RalB-1: 5′-TGACGAGTTTGTAGAAGACdTT-3′
RalB-2: 5′-CAAAGACGTGATGAGTTAATTdTT-3′
Beclin1-1: 5′-ACCGACTTGTTCCTTACGGAAdTT-3′
Beclin1-2: 5′-TGGACAGTTTGGCAATCAAdTT-3′
ATG5-2: 5′ AACCTTTGGCCTAAGAAGAAAdTT-3′
Sec5: 5′-GGTCGGAAAGACAAGGCAGdTT-3′
Exo84: 5′-CCACTTTACTCTATATTCAdTT-3′
Parkin1-1: 5′-CAGTTTGTTCACGACCCTCAAdTT-3′
Parkin1-2: 5′-CAGAGGAAAGTCACCTGCGAAdTT-3′
PINK1-1: 5′-CAAGTCCGACAACATACTTdTT-3′
PINK1-2: 5′-TAACTGCAGGCCAACACGCdTT-3′
USP30: 5′-GTGACAACTGTACAAAGATTGdTT-3′
p21/Cip1: 5′-ATAATTAAGACACACAAACdTT-3′

### Cell proliferation and cell viability analyses

Cells were seeded at 8 × 10 E4 cells per well in 6-well plates and at indicated time points counted using the automated ViCell-XR cell counter (Beckman Coulter). Cell viability was determined by trypan blue exclusion.

### Soft agar assays for anchorage-independent growth

After trypsinization, cells were passed 4-5 times through a 21G syringe, before 1 × 10 E4 cells were resuspended in complete medium with 0.3% agarose (A2576, Sigma) and appropriate antibiotics, and subsequently cultured in 15 ml tubes (352059, BD Biosciences) overlaid with medium without agarose. After two weeks, agarose gels were very gently poured into wells of 6-well plates, colonies stained with 2.5 mg/ml MTT (Sigma), scanned, and finally quantified using the Image J software. Cell clusters of at least 50 cells were scored as colonies.

### Xenograft animal experiments

5 × 10 E6 exponentially growing cells were subcutaneously injected into the interscapular fat pad of six weeks-old female Swiss nude mice (Charles Rivers Laboratories, France). Tumour growth was evaluated by measuring two perpendicular diameters of tumours with a caliper three times per week. Individual tumour volumes were calculated as *V* = *a* × *b*^2^/2, with *a* being the major diameter, and *b* the minor diameter. Tumour growth curves were established as a function of time. Mice were sacrificed once tumours reached a volume of 1500 mm^3^. Experiments were performed in compliance with animal welfare regulations of the European Community (86/609/EEC), the French Competent Authority, and the UKCCCR guidelines.

### Cell cycle, apoptosis and mitochondrial parameter measurements by flow cytometry

To determine cell cycle profiles, cells were fixed in ice cold 70% EtOH/PBS, washed with PBS and subsequently incubated in PBS containing 50 mg/ml propidium iodide (PI, Sigma) and 500 units/ml RNaseA (Life Technologies). To measure apoptotic cells, cells were stained with AnnexinV-FITC antibody and PI according to manufacturer's instructions (BD Pharmingen). To assess mitochondrial mass, cells were stained with the mitochondrial membrane potential-independent MitoTracker Green FM (Invitrogen) as instructed by the manufacturer. Mitochondrial membrane potential (ΔΨm) was tested by staining cells with JC-1 (Invitrogen) according to manufacturer's instructions. ΔΨm is expressed as the ratio between JC-1 red fluorescence and JC-1 green fluorescence. The JC-1 dye exhibits potential-dependent accumulation in mitochondria, indicated by a fluorescence emission shift from green (~529 nm, representing depolarized mitochondria) to red (~590 nm, indicating polarized mitochondria). Mitochondrial depolarization is indicated by a decrease in the red/green fluorescence intensity ratio. Mean Fluorescence Intensity (MFI) was measured by flow cytometry. All samples were analysed using the LSRII flow cytometer (BD Biosciences).

### Immunofluorescence microscopy, immunoblotting, pull down experiments, and densitometry analysis

Immunofluorescence (IF) and Western blot analyses were performed as defined [29]. IF images were acquired using a Zeiss Axiovert 200M Apotome microscope. For LB3B puncta quantifications, fields were chosen arbitrarily based on DAPI staining and the number of LC3 dots per cell was determined using Image J software. Immunoblot signals were visualized by enhanced chemiluminescence detection (Western Lightning Plus-ECL, PerkinElmer). Densitometric analyses of immunoblots were performed using the Multi Gauge software (FujiFilm). Pull downs were performed as reported [59] using the Ral binding domain (RalBD) of Sec5 (residues 1 to 99) fused to GST as Ral-GTP trap. Briefly, cells were lysed in lysis buffer (50 mM Tris pH 7.4, 150 mM NaCl, 10 mM MgCl2, 1% Triton X-100, 0.1% SDS, freshly supplemented with a cocktail of proteases inhibitors and 1 mM DTT). Subsequently, lysates were incubated with GST-RalBD bound to glutathione-Sepharose for 40 minutes at 4°C, before washing with lysis buffer, and final analysis by Western blotting.

### Reactive oxygen species (ROS) measurements by flow cytometry

To assess intracellular levels of ROS, cells were stained with the Carboxy-H_2_DCFDA dye (Invitrogen) according to manufacturer's instructions. To assess mitochondrial ROS levels, cells were stained with MitoSOX Red (Invitrogen) according to manufacturer's instructions.

### Antibodies

For Western blotting the following primary antibodies were used: STK38 (H00011329-M011, Abnova), Cleaved-PARP (552597), Adaptin (610502), RalA (610222), and Ras (610002) from BD Biosciences; Beclin1 (3495), LC3B (2775), ATG5 (2630), RalB (3523) p21/Cip1 (2947), pAKT (9271), AKT (4685), pERK (4370), and ERK (4695) from Cell signalling; PINK1 (ab23707), Parkin (ab15954), TOM20 (ab56783) and the total OXPHOS antibody cocktail (ab110411) from Abcam; HA (12CA5, Roche); β-Actin (A5441, Sigma-Aldrich); and GAPDH (MAB374, Millipore). Polyclonal rabbit anti-T444-P and anti-STK38L antibodies have been described [28, 31, 60]. Rabbit polyclonal antibodies raised against Sec5 and Exo84 were kind gifts from Charles Yeaman (University of Iowa) [61]. Donkey anti-rabbit-HRP and anti-mouse-HRP secondary antibodies were from Jackson Immunoresearch and GE Healthcare, respectively. For immunofluorescence we used anti-LC3B (5F10, nanoTools) as primary antibody and anti-mouse Alexa488 (Jackson ImmunoResearch) as secondary antibody.

### RNA isolation, cDNA synthesis, and real-time quantitative PCR gene expression analysis

Total RNAs were extracted from cells at 80% – 90% confluence using the RNeasy Plus Mini Kit (Qiagen). cDNA synthesis was performed using the iScript cDNA synthesis Kit (Bio-Rad) according to manufacturer's protocol. qPCR was carried out in triplicates using validated qPCR primers (Qiagen; USP30, Hs.486434; STK38, Hs.409578; Sec5, Hs.484412; and GAPDH, Hs.544577) and the TaqMAN PCR master mix (Applied Biosystems) using the Chromo4 DNA Engine Peltier Thermal Cycler (Bio-Rad). GAPDH mRNA served as internal control for standardization.

### Plasmids

Retroviral plasmids encodingHRasG12V, HRasG12V/E37G, HA-STK38 PIF, HA-STK38 PIF/kd, and shLUC have been reported [34, 38, 62]. To generate the pSuper.retro.puro_shSTK38-2 vector expressing shRNAs against human STK38, the following oligonucleotide pairs were inserted into pSuper.retro.puro (Oligoengine) using *BglII* and *HindIII*: 5′-GATCCCCGTCGGCCATAAACAGCTATTCAAGAGATAGCTGTTTATGGCCGACGTTTTTGGAAA-3′ and 5′- AGCTTTTCCAAAAACGTCGGCCATAAACAGCTATCTCTTGAATAGCTGTTTATGGCCGACGGG-3′ targeting the 3′UTR of STK38. For shRNA rescue experiments, HA-tagged STK38 wild-type and kinase-dead (K118A) cDNAs were subcloned from modified pcDNA3_HA into modified pWZLblast using *Pme*I and *Xho*I. Modified pcDNA_HA was generated by inserting the following oligonucleotide pairs into pcDNA3 (Invitrogen) using *Hind*III and *BamH*I: 5′-AGCTTACGCGTGTTTAAACGGTACCATGGCCTACCCCTACGACGTGCCCGACTACGCCTCCCTCG-3′ and 5′-GATCCGAGGGAGGCGTAGTCGGGCACGTCGTAGGGGTAGGCCATGGTACCGTT TAAACACGCGTA-3′. Modified pWZLblast was generated by inserting the following oligonucleotide pairs into pWZLblast GFP (12269, Addgene) using *BamH*I and *Sal*I: 5′-GATCCACGCGTGTTTAAACGTTAACGAATTCTACGTACTCGAGG -3′ and 5′-TCGACCTCGAGTACGTAGAATTCGTTAACGTTTAAACACGCGTG -3′. All constructs were confirmed by sequence analysis. Further details on constructs are available upon request.

### Statistical analysis

Unless otherwise stated the significance of differences between the means or the population distributions was determined using two-tailed unpaired Student's *t*-tests. For tumour volume measurements and Ral-GTP pulldown assays, the *p*-values were calculated using the Mann-Whitney statistical test. For all tests, differences were considered statistically significant when *p*-values were below 0.05 (*), 0.01 (**), or 0.001 (***). In the Figures *p*-values are indicates as: *p<0.05; **p<0.01; ***p<0.001; ns, not significant. Error bars represent standard deviation.

## SUPPLEMENTARY FIGURES


