# Correction: Mitochondrial clearance by the STK38 kinase supports oncogenic Ras-induced cell transformation

**DOI:** 10.18632/oncotarget.25335

**Published:** 2018-04-27

**Authors:** Audrey Bettoun, Carine Joffre, Giulia Zago, Didier Surdez, David Vallerand, Ramazan Gundogdu, Ahmad A.D. Sharif, Marta Gomez, Ilaria Cascone, Brigitte Meunier, Michael A. White, Patrice Codogno, Maria Carla Parrini, Jacques H. Camonis, Alexander Hergovich

**Affiliations:** ^1^ Institut Curie, Inserm U830, Paris Sciences et Lettres University Paris,75248, France; ^2^ University College London, Cancer Institute, London, WClE 6BT, United Kingdom; ^3^ University of Texas Southwestern Medical Center, Dallas, Texas, 75390, USA; ^4^ Inserm U1151-CNRS UMR 8253, Institut Necker Enfants-Malades, Paris, 75993, France; ^5^ Present address: Cancer Research Center of Toulouse, UMR1037, Toulouse, 31100, France

**This article has been corrected:** The correct Grant Support information is given below:

**GRANT SUPPORT**

This work was supported by the Wellcome Trust Research Career Development fellowship 090090/Z/09/Z (to A Hergovich); the BBSRC grant BB/102124811 (to A. Hergovich); the National Institute for Health Research University College London Hospitals Biomedical Research Centre; the Ministry of National Education of the Republic of Turkey (to R. Gundogdu); the Ligue Nationale contre le cancer (LNCC) grants RD14/75-80 (to J. Camonis) and RS14/75-54 (to M.C. Parrini); the Fondation ARC grant SFI20121205710 (to M.C. Parrini); the ARC grant SFI20111203931 (to J. Camonis); the ANR grant ANR-13-CESA-0010-001 (to P. Codogno); the Association Christelle Bouillot; INCA; and institutional INSERM and CNRS funding. Work was suported also by grantANR-08-NLAN-0290-01 to J. Camonis.

Original article: Oncotarget. 2016; 7:44142-44160. https://doi.org/10.18632/oncotarget.9875

