# Supplementary material for: Mitochondrial clearance by the STK38 kinase supports oncogenic Ras-induced cell transformation
Source: Oncotarget. 2016 Jun 7;7(28):44142–60. doi: 10.18632/oncotarget.9875 (PMC5190085; doi:10.18632/oncotarget.9875)
Supplement: Supplementary file 1 [file oncotarget-07-44142-s001.pdf]

Mitochondrial clearance by the STK38 kinase supports oncogenic Ras-induced cell transformation

SUPPLEMENTARY FIGURES

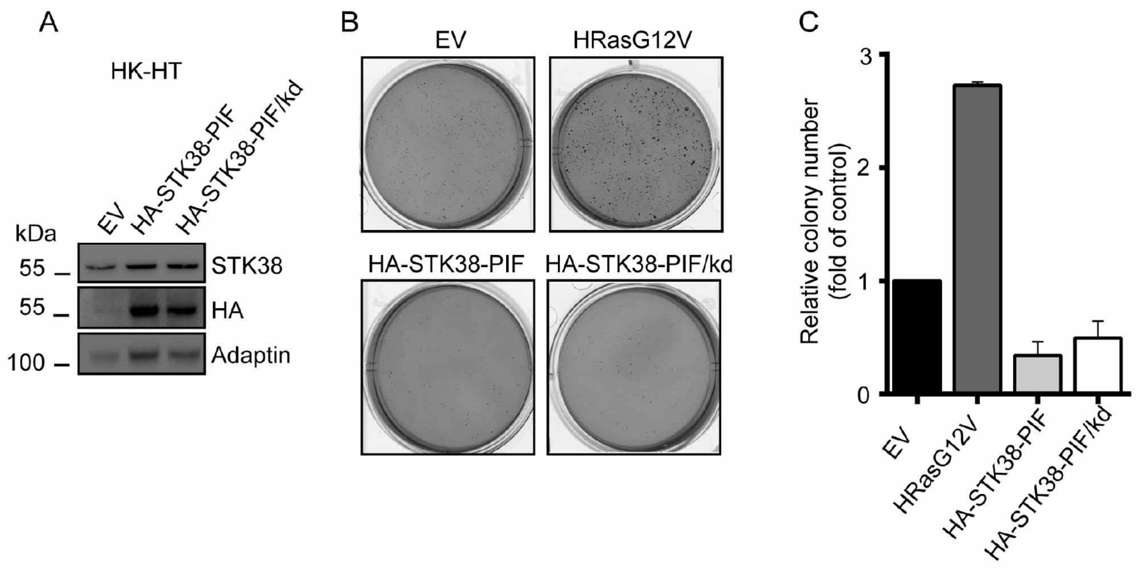

Supplementary Figure S1 (in support of Figure 1): Constitutively active NDR1 is not sufficient to promote anchorage-independent growth of HK-HT cells. A. Immunoblotting with indicated antibodies of cell lysates derived from HK-HT cells stably expressing empty vector (EV), HA-tagged constitutively active NDR1 (HA-NDR1-PIF), or HA-tagged kinase-dead NDR1 (HA-NDR1-PIF/kd). **B.** Cells stably expressing indicated cDNAs were subjected to soft agar growth assays. Representative images of soft agar assays are shown. **C.** Histograms represent the quantification of colony formation in soft agar. The average of two independent experiments performed in duplicates is shown.

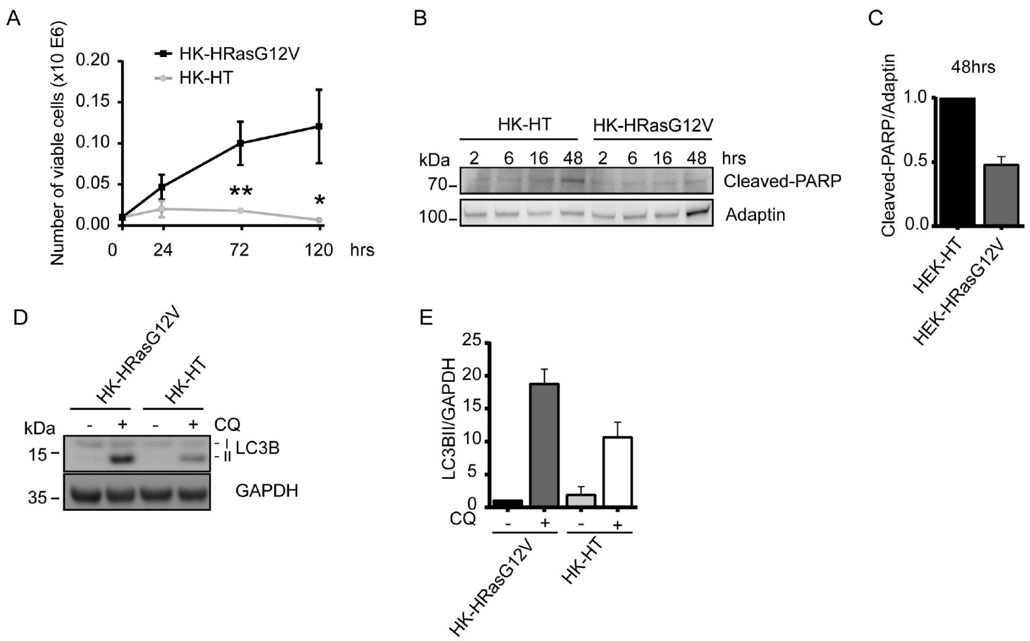

Supplementary Figure S2 (in support of Figures 3 and 4): Expression of oncogenic HRas^{G12V} in HK-HT results in anoikis resistance and increased detachment-induced autophagy. A. Graph comparing the proliferation rates of HK-HT and HK-HRasG12V cells grown in suspension. The average of three independent experiments performed in duplicates is shown (n=3, *p<0.05; **p<0.01). **B, C.** Immunoblot of HK-HT and HK-HRasG12V cells grown in suspension for indicated times before processing for Western blotting. One representative of two independent experiments is shown (B). Histograms show the densitometry quantification of immunoblots from two independent experiments (C). **D, E.** Immunoblot analysis of HK-HT and HK-HRasG12V cells grown in suspension for 16 hrs with (+) or without (-) chloroquine (CQ). Lipidated (LC3B-II) and non-lipidated (LC3B-I) LC3B is indicated. One representative of two independent experiments is shown (D). Histograms show the densitometry quantification of immunoblots (E).

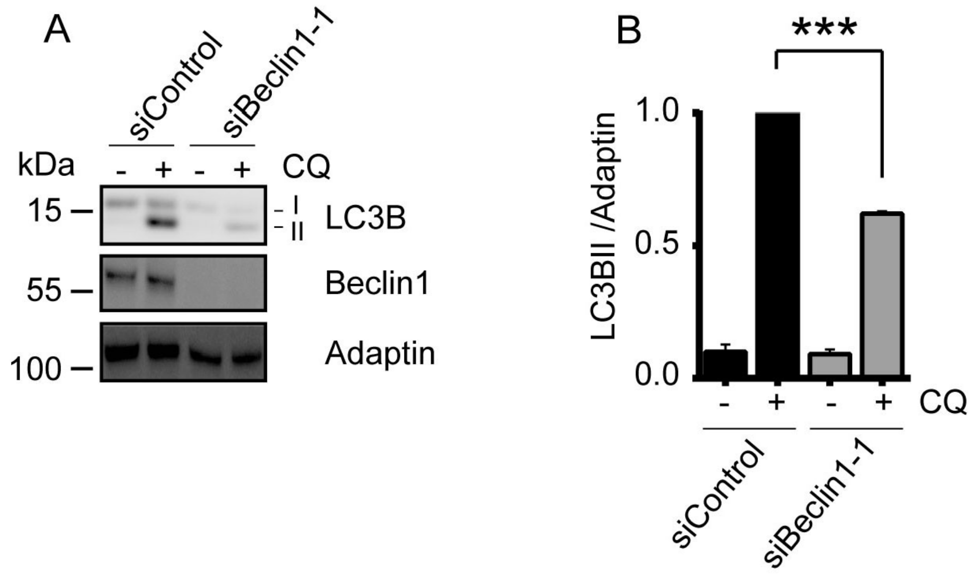

Supplementary Figure S3 (in support of Figure 4): Beclin1 depletion impairs detachment-induced autophagy in HK-HRasG12V cells. A. Immunoblot analysis of HK-HRasG12V cells transiently transfected with indicated siRNAs and grown in suspension for 16 hrs with (+) or without (-) chloroquine (CQ). Lipidated (LC3B-II) and non-lipidated (LC3B-I) LC3B is indicated. One of three independent experiments is shown. **B.** Histograms show the densitometry quantification of immunoblots from three independent experiments (n=3, ***p<0.001).

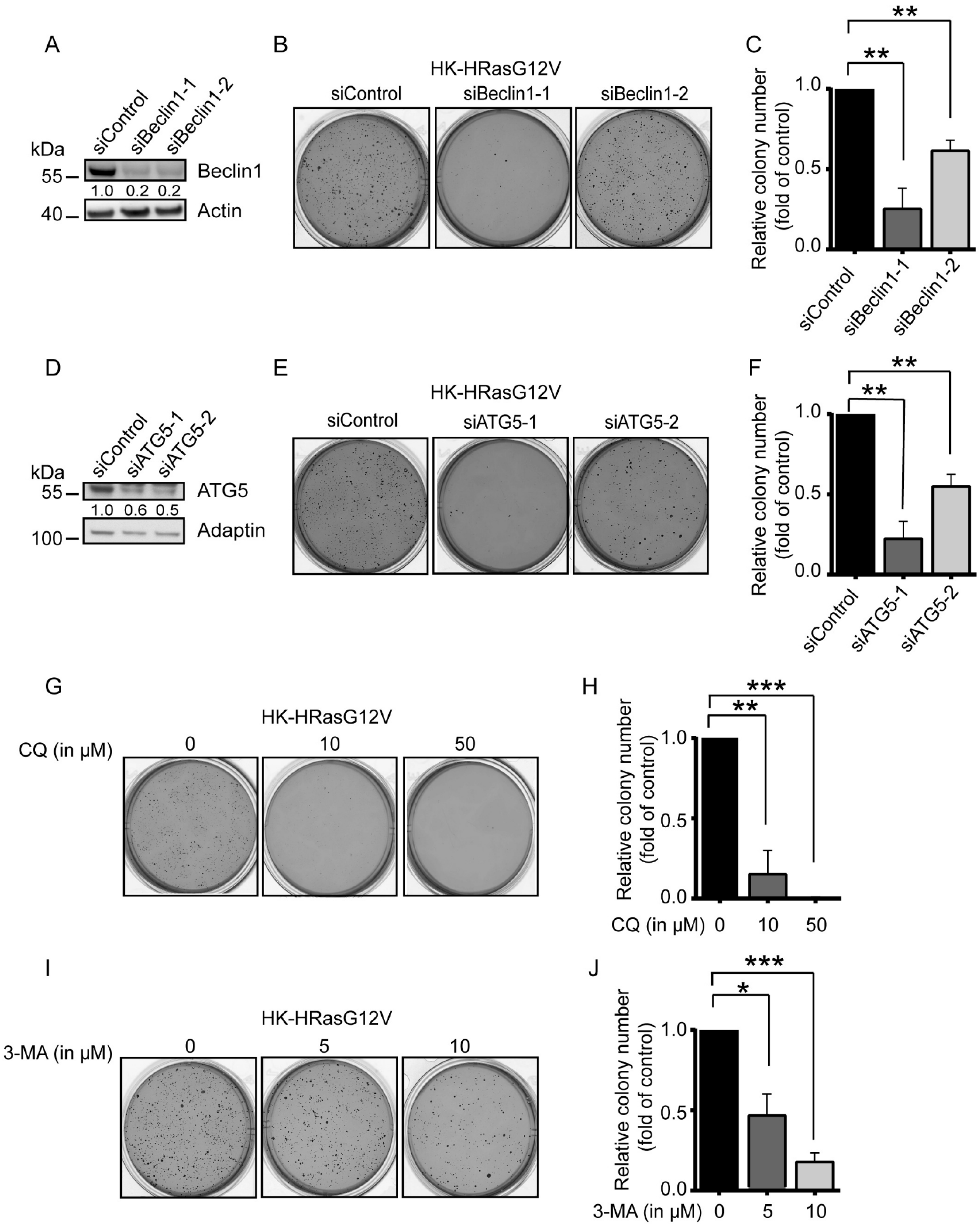

Supplementary Figure S4 (in support of Figure 4): Autophagy is required for anchorage independent growth of HK-HRasV12 cells. A, D. Immunoblotting with indicated antibodies of cell lysates derived from HK-HRasG12V cells transiently transfected for 72 hours (hrs) with indicated siRNAs. Densitometry quantifications of immunoblots are indicated below the immunoblots. **B, C, E, F.** Depletion of Beclin1 or ATG5 significantly decreases anchorage-independent growth of HK-HRasG12V cells. Cells transiently transfected with indicated siRNAs were subjected to soft agar growth assays. Representative images of soft agar assays are shown (B, E). Histograms represent the quantification of colony formation in soft agar (C, F). The average of three independent experiments performed in duplicates is shown (n=3, $^{**}p<0.01$). **G-J.** Pharmacolgical inhibition of autophagy diminishes anchorage-independent growth of HK-HRasG12V cells. Cells were grown in soft agar medium containing the indicated doses of chloroquine (CQ) or pre-treated overnight with 3-Methyladenine (3-MA) before plating in soft agar. Representative images of soft agar assays are shown (G, I). Histograms represent the quantification of colony formation in soft agar (H, J). The average of three independent experiments performed in duplicates is shown (n=3, $^{*}p<0.05$; $^{**}p<0.01$; $^{***}p<0.001$).

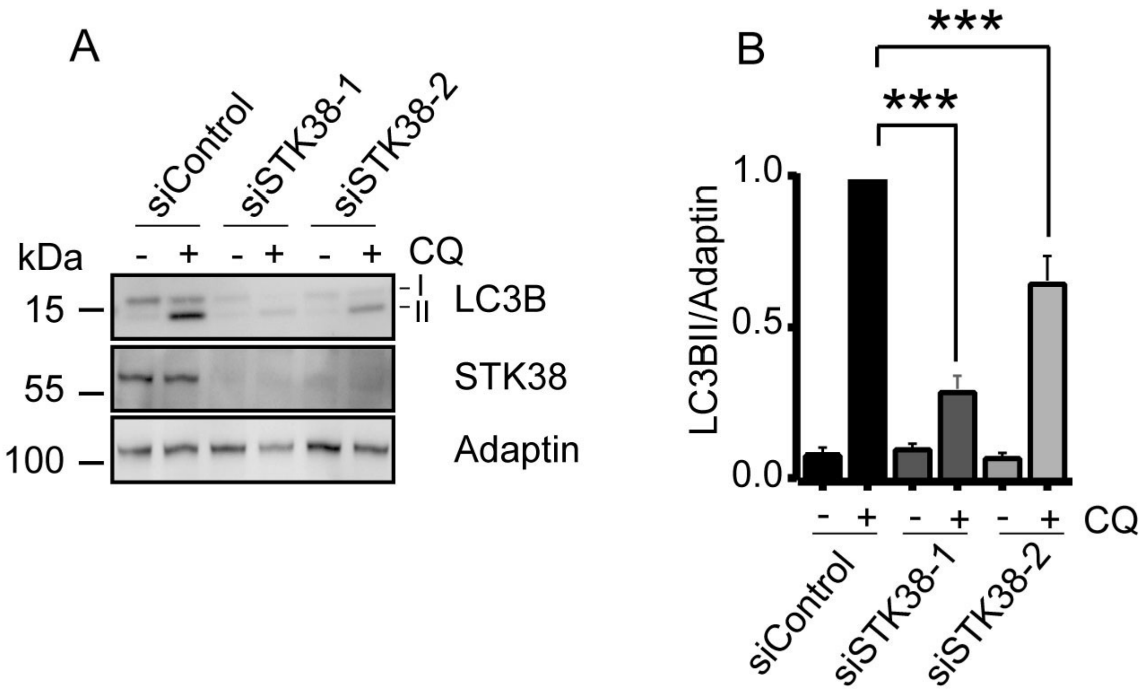

Supplementary Figure S5 (in support of Figure 4): Depletion of NDR1 with independent siRNAs significantly impacts detachment-induced autophagy in HK-HRasG12V cells. A. Immunoblot analysis of HK-HRasG12V cells transiently transfected with indicated siRNAs and grown in suspension for 16 hrs with (+) or without (-) chloroquine (CQ). Lipidated (LC3B-II) and non-lipidated (LC3B-I) LC3B is indicated. One of three independent experiments is shown. **B.** Histograms show the densitometry quantification of immunoblots from three independent experiments (n=3, ****p*<0.001).

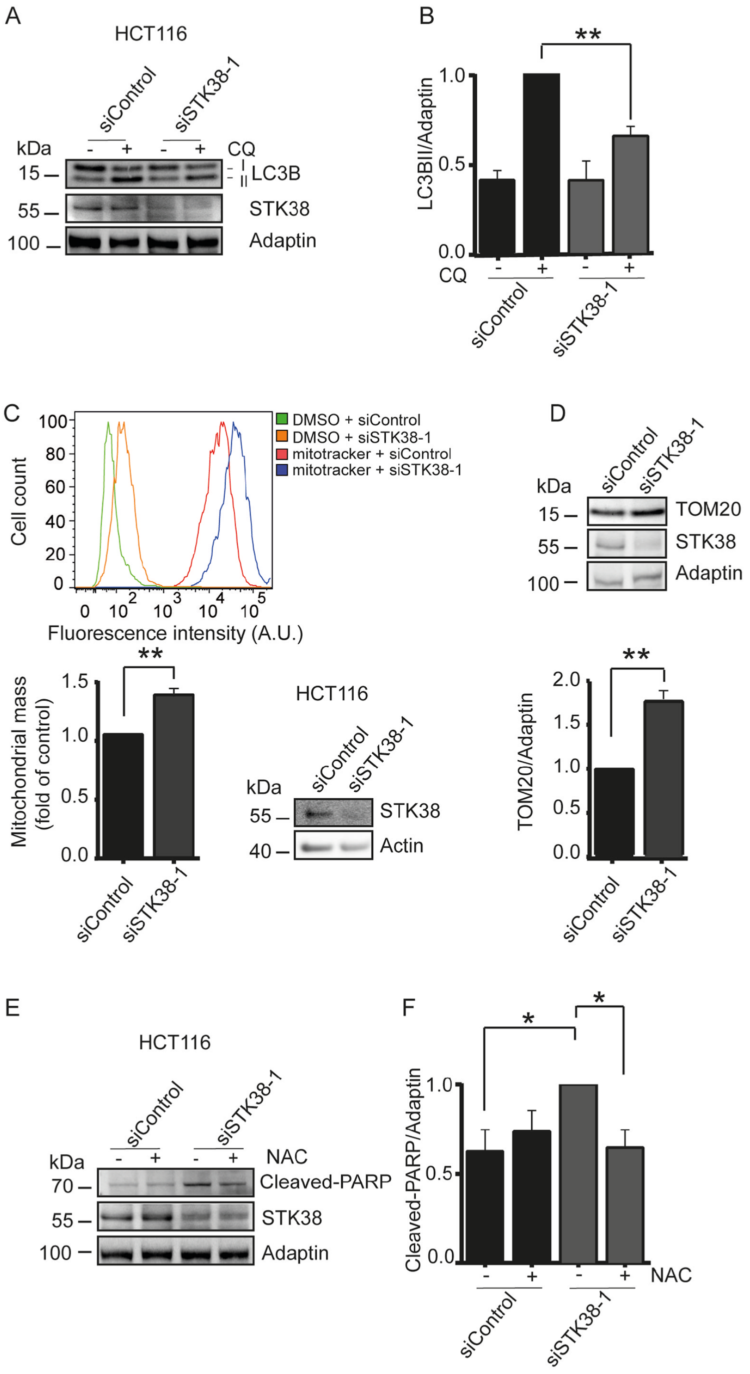

Supplementary Figure S6 (in support of Figures 4 and 6): STK38 supports detachment-induced autophagy and mitophagy to prevent ROS-mediated anoikis in detached HCT116 cells. A, B. Immunoblot analysis of HCT116 cells transiently transfected with indicated siRNAs and grown in suspension conditions with (+) or without (-) chloroquine (CQ) (A). Lipidated (LC3B-II) and non-lipidated (LC3B-I) LC3B is indicated (A). One of three experiments is shown. Densitometry quantification of immunoblots (B, n=3, $**p<0.01$). **C, D.** HCT116 cells transiently transfected with indicated siRNAs and grown in suspension were subjected to flow cytometry (C) and immunoblotting (D) using indicated dye and antibodies. One of three experiments is shown (top panels). Quantifications of total mitochondrial mass (C) and densitometry of immunoblots (D) (bottom panels) (n=3, $**p<0.01$). Validation of STK38 knockdown is shown as insert (C). **E, F.** HCT116 cells were transiently transfected with indicated siRNAs and subsequently grown in suspension in the absence (-) or presence (+) of N-acetylcysteine (NAC), followed by processing for immunoblotting. One of three experiments is shown (E). Quantifications of densitometry of immunoblots (F, n=3, $*p<0.05$).

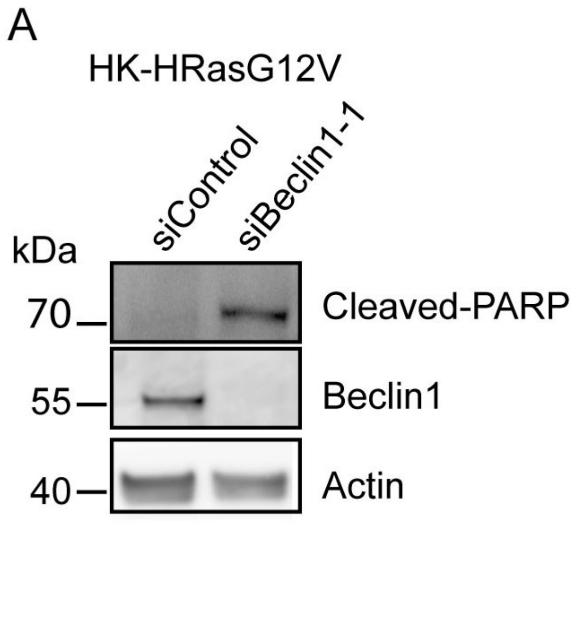

Supplementary Figure S7 (in support of Figure 4): Depletion of Beclin1 causes decreased anoikis resistance of HK-HRasG12V cells. A. Immunoblot of cells transiently transfected with indicated siRNAs and grown in suspension for 16 hrs. One of three independent experiments is shown. **B.** Histograms show the densitometry quantification of immunoblots from three independent experiments (n=3, ***p*<0.01).

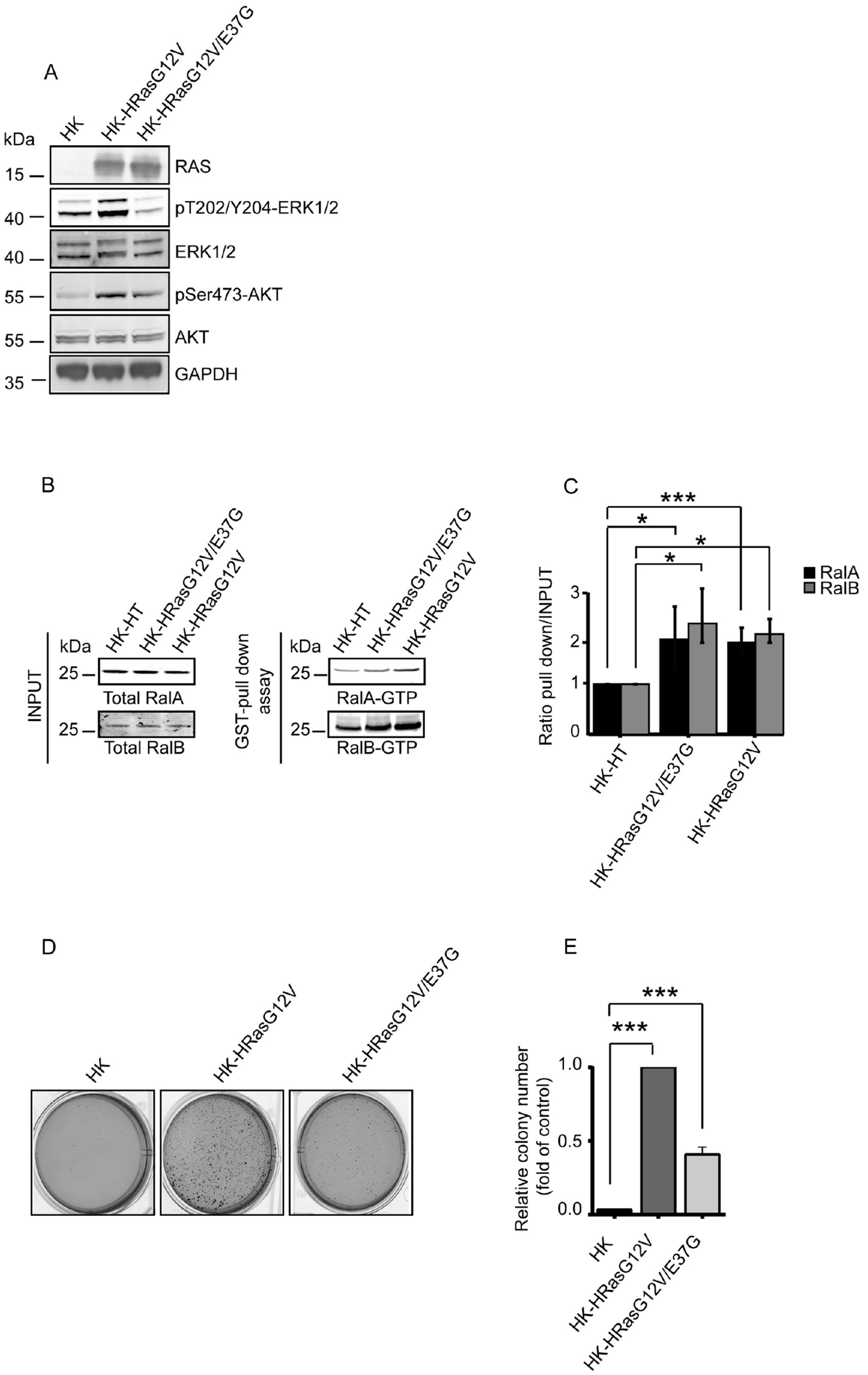

Supplementary Figure S8 (in support of Figure 5): Ras-Ral signalling is sufficient to promote anchorage-independent growth of HK-HT cells. A. Immunoblotting with indicated antibodies of cell lysates derived from HK-HT cells stably expressing empty vector (EV), $HRas^{G12V}$, or $HRas^{G12V/E37G}$. Cells were grown in suspension for 16 hrs prior to lysis. HEK-HT-HRasG12V/E37G cells displayed diminished Raf and PI3K signaling as compared to HEK-HT-HRasG12V cells. **B, C.** To determine RalA and RalB activation by $HRas^{G12V}$ or $HRas^{G12V/E37G}$, cell lysates of the indicated cell lines grown in suspension were subjected to Ral-GTP pulldowns using GST-RalBD as bait, followed by Western blotting (B). Quantifications of immunoblots are shown (C, n=3, *p<0.05; ***p<0.001). GST alone did not show any specific binding to RalA or RalB (not shown). **D.** Cells stably expressing indicated cDNAs were subjected to soft agar growth assays. Representative images of soft agar assays are shown. **E.** Histograms represent the quantification of colony formation in soft agar. The average of three independent experiments performed in duplicates is shown (n=3, ***p<0.001).

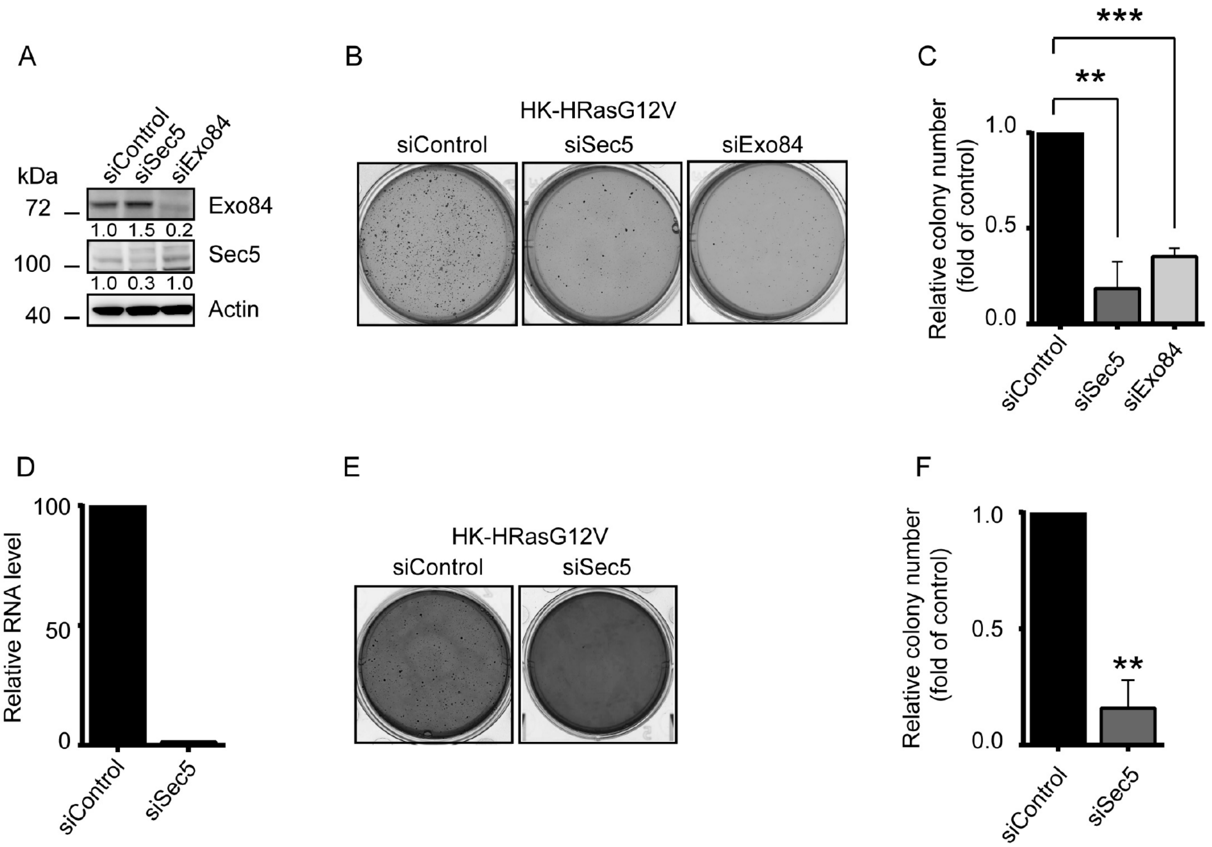

Supplementary Figure S9 (in support of Figure 5): The Exocyst components Sec5 and Exo84 are required for anchorage-independent growth of HK-HRasG12V cells. A. Western blotting of HK-RasG12V cells transiently transfected with indicated siRNAs. Densitometry quantifications of immunoblots are indicated below the immunoblots. **B.** Cells transiently expressing indicated siRNAs were subjected to soft agar growth assays. Representative images of soft agar assays are shown. **C.** Histograms represent the quantification of colony formation in soft agar. The average of three independent experiments performed in duplicates is shown (n=3, ***p*<0.01; ****p*<0.001). **D.** Quantitative RT-PCR analysis of Sec5 mRNA levels of HK-RasG12V cells transiently transfected with indicated siRNAs. **E.** Cells transiently expressing indicated siRNAs were subjected to soft agar growth assays. Representative images of soft agar assays are shown. **F.** Histograms represent the quantification of colony formation in soft agar. The average of three independent experiments performed in duplicates is shown (n=3, ***p*<0.01).

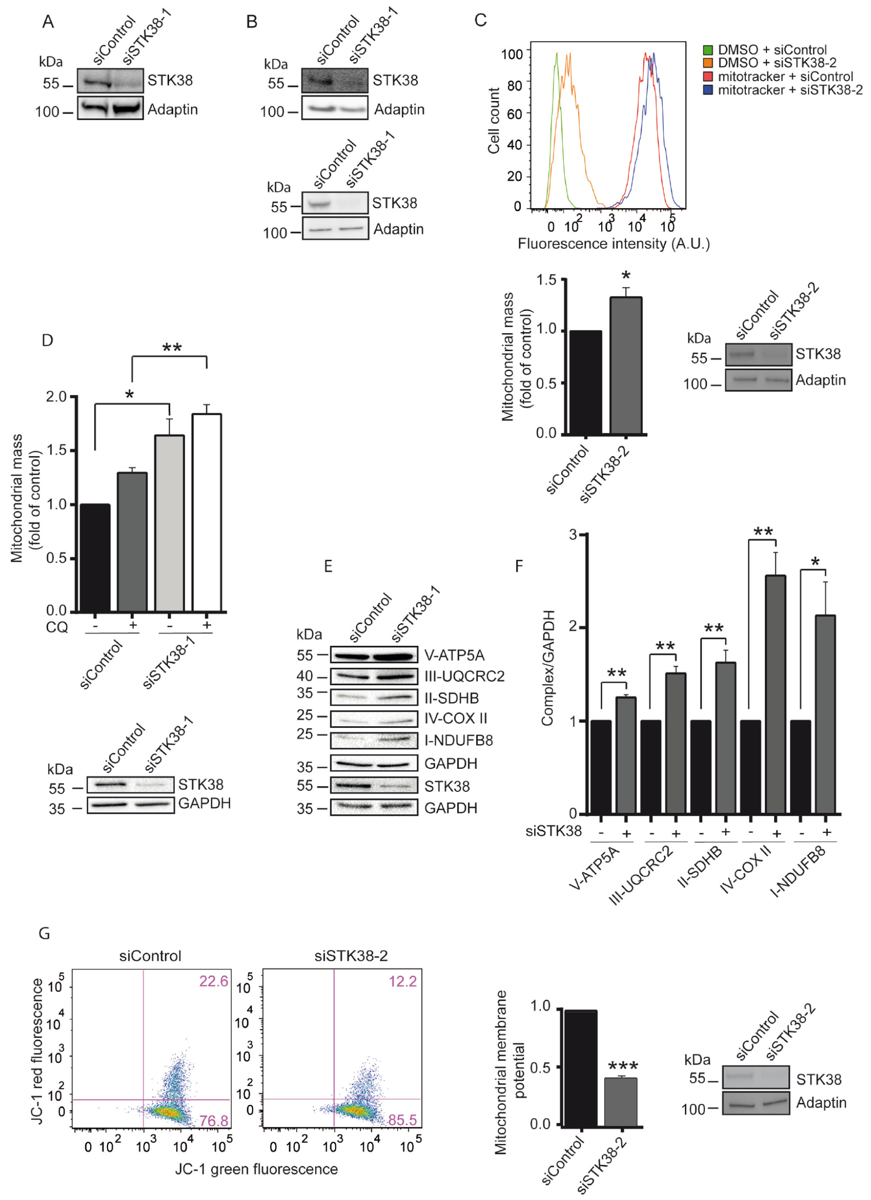

Supplementary Figure S10 (in support of Figure 6): NDR1 depletion results in increased total mitochondrial mass and decreased mitochondrial membrane potential in Ras-transformed human cells. A, B. Validation of NDR1 knockdown in support of Figure 6A (A), Figure 6D (B, top panel) and Figure 6E (B, bottom panel). Immunoblots of HK-HRasG12V cells transiently transfected with indicated siRNAs and grown in suspension for 16 hrs before cell lysis. **C.** Cells transiently transfected with indicated siRNAs and grown in suspension were subjected to flow cytometry using a membrane potential-independent MitoTracker, which visualises the total mitochondrial mass. One of three independent experiments is shown (top panels). Histograms (bottom panels) show the quantifications of total mitochondrial mass from three experiments (n=3, *$p<0.05$). Validation of NDR1 knockdown by immunoblotting is shown as insert. **D.** Cells transiently transfected with indicated siRNAs and grown in suspension in the absence or presence of chloroquine (CQ) were subjected to flow cytometry using a membrane potential-independent MitoTracker. Histograms (top panel) show the quantifications of total mitochondrial mass n=3, *$p<0.05$; **$p<0.01$. Validation of NDR1 knockdown by immunoblotting is shown as insert (bottom panel). **E, F.** Cells transiently transfected with indicated siRNAs and grown in suspension were subjected to immunoblotting using indicated antibodies. One of three experiments is shown (E). Quantifications of densitometry of immunoblots are shown as histograms (F, n=3, *$p<0.05$; **$p<0.01$). **G.** Cells transiently transfected with indicated siRNAs and grown in suspension were processed for flow cytometry using the JC-1 dye, which visualises the mitochondrial membrane potential. One of three independent experiments is shown (left panels). Histograms (middle panel) show the quantification of mitochondrial membrane potential from three independent experiments (n=3, ***$p<0.001$). Validation of NDR1 knockdown by immunoblotting is shown as insert (right panel).

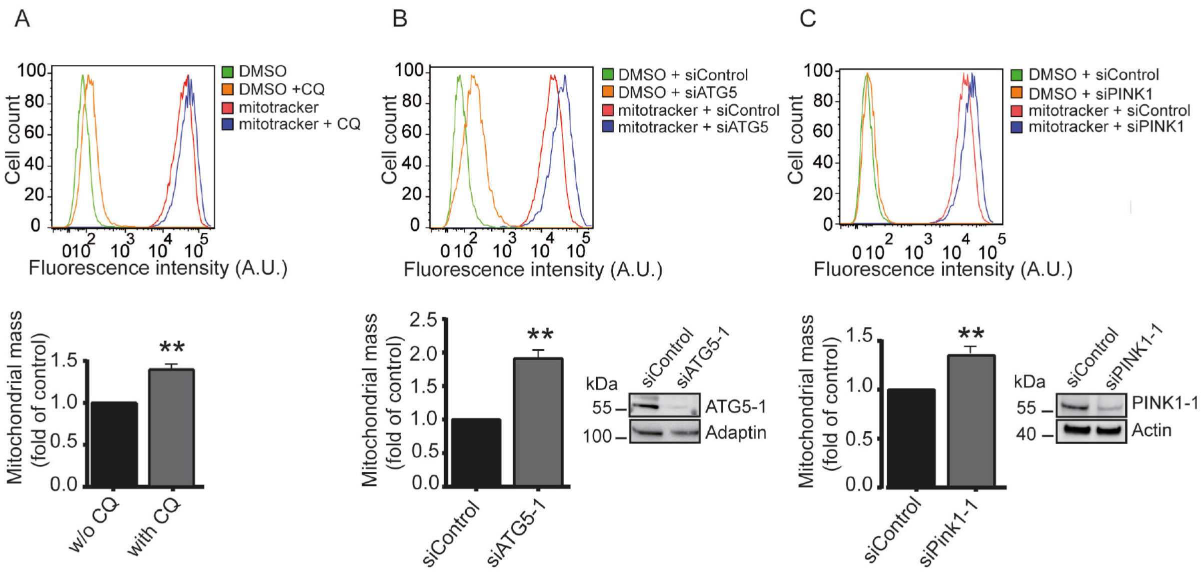

Supplementary Figure S11 (in support of Figure 6): Pharmacological/genetic inhibition of autophagy and PINK1 depletion results in increased total mitochondrial mass in detached Ras-transformed human cells. HK-HRasG12V cells were grown in suspension with or without chloroquine (CQ) before processing for flow cytometry to determine total mitochondrial mass **A.** Likewise, ATG5- or PINK1-depleted cells were grown in suspension, followed by flow cytometry analysis to determine total mitochondrial mass **B, C.** One of three experiments is shown (top panels). Quantifications of total mitochondrial mass (n=3, ***p*<0.01). Validations of ATG5 and PINK1 knockdown are shown as inserts.

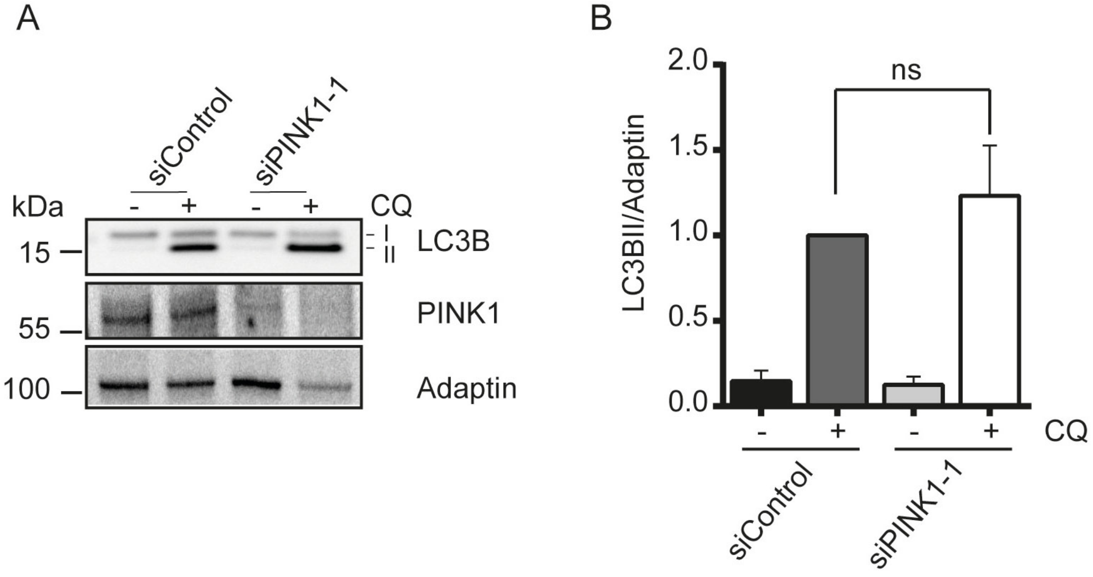

Supplementary Figure S12 (in support of Figure 6): PINK1-depleted HK-HRasG12V cells display normal detachment-induced autophagy as judged by LC3B-II conversion assays. Lysates of HK-HRasG12V cells transiently transfected with indicated siRNAs and grown in suspension with or without CQ were analysed by quantitative immunoblotting **A.** Lipidated (LC3B-II) and non-lipidated (LC3B-I) LC3B is indicated (A). One representative result is shown. Histograms show the densitometry quantification of immunoblots from three independent experiments **B.** (n=3; ns, not significant).

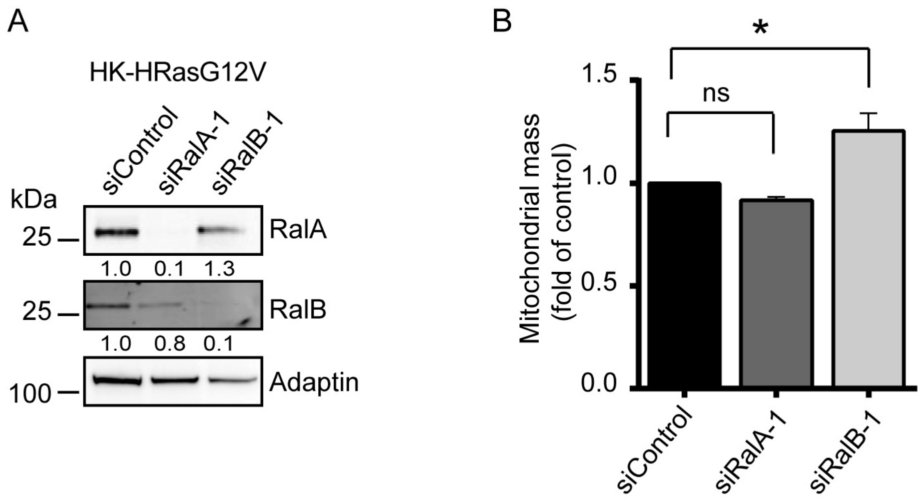

Supplementary Figure S13 (in support of Figure 6): RalB depletion impairs mitophagy in HK-HRasG12V cells, while RalA knockdown has no significant impact. RalB depletion, but not RalA knockdown, in HK-HRasG12V cells results in increased mitochondrial mass. Cells transiently transfected with indicated siRNAs and grown in suspension for 16 hrs were subjected to immunoblotting **A.** and flow cytometry **B.** using indicated antibodies and a membrane potential-independent MitoTracker, respectively. Densitometry quantifications of immunoblots are indicated below the immunoblots (A). Quantifications of the total mitochondrial mass from three independent experiments (B, n=3, **p*<0.05; ns, not significant).

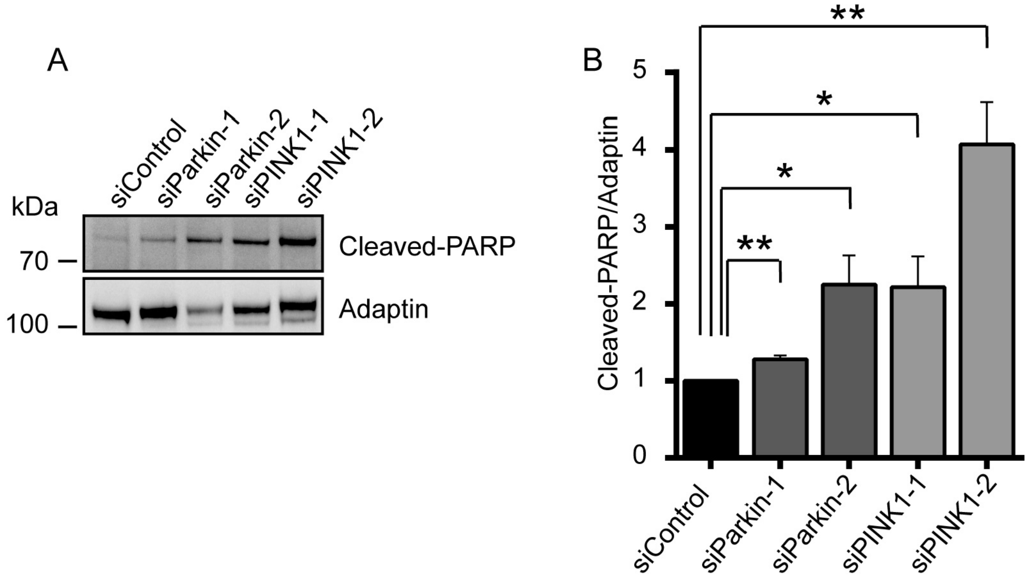

Supplementary Figure S14 (in support of Figure 7): The mitophagy regulators Parkin and PINK1 support anoikis resistance of Ras-transformed human cells. Immunoblot analysis of HK-RasG12V detached cells transiently transfected with indicated siRNAs. Cells were grown in suspension for 16 hrs before processing for immunoblotting. One of three independent experiments is shown **A.** Histograms show the densitometry quantification of immunoblots from three independent experiments **B.** (n=3, **p*<0.05; ***p*<0.01).
